# Tropomodulin3 is a novel Akt2 effector regulating insulin-stimulated GLUT4 exocytosis through cortical actin remodeling

**DOI:** 10.1038/ncomms6951

**Published:** 2015-01-09

**Authors:** Chun-Yan Lim, Xuezhi Bi, Donghai Wu, Jae Bum Kim, Peter W. Gunning, Wanjin Hong, Weiping Han

**Affiliations:** 1 Laboratory of Metabolic Medicine, Singapore Bioimaging Consortium, Agency for Science, Technology and Research, Singapore 138667, Singapore; 2 Bioprocessing Technology Institute, Agency for Science, Technology and Research, Singapore 138668, Singapore; 3 The Key Laboratory of Regenerative Biology and The Guangdong Provincial Key Laboratory of Stem Cell and Regenerative Medicine, Guangzhou Institute of Biomedicine and Health, Chinese Academy of Sciences, Guangzhou 510530, China; 4 National Creative Research Initiatives Center for Adipose Tissue Remodeling, Department of Biological Sciences, Institute of Molecular Biology and Genetics, Seoul National University, Seoul 151-742, Republic of Korea; 5 Oncology Research Unit, School of Medical Sciences, University of New South Wales, Sydney, New South Wales 2052, Australia; 6 Institute of Molecular and Cell Biology, Agency for Science, Technology and Research, Singapore 138673, Singapore

## Abstract

Akt2 and its downstream effectors mediate insulin-stimulated GLUT4-storage vesicle (GSV) translocation and fusion with the plasma membrane (PM). Using mass spectrometry, we identify actin-capping protein Tropomodulin 3 (Tmod3) as an Akt2-interacting partner in 3T3-L1 adipocytes. We demonstrate that Tmod3 is phosphorylated at Ser71 on insulin-stimulated Akt2 activation, and Ser71 phosphorylation is required for insulin-stimulated GLUT4 PM insertion and glucose uptake. Phosphorylated Tmod3 regulates insulin-induced actin remodelling, an essential step for GSV fusion with the PM. Furthermore, the interaction of Tmod3 with its cognate tropomyosin partner, Tm5NM1 is necessary for GSV exocytosis and glucose uptake. Together these results establish Tmod3 as a novel Akt2 effector that mediates insulin-induced cortical actin remodelling and subsequent GLUT4 membrane insertion. Our findings suggest that defects in cytoskeletal remodelling may contribute to impaired GLUT4 exocytosis and glucose uptake.

## Abstract (web summary)

Insulin-stimulated GLUT4-storage vesicle fusion with the plasma membrane depends on activity of Akt2 and its downstream effectors. Here Lim *et al.* identify the actin-capping protein tropomodulin 3 as a novel Akt2 effector that remodels cortical actin to help facilitate GLUT4 membrane insertion.

## Graphical abstract

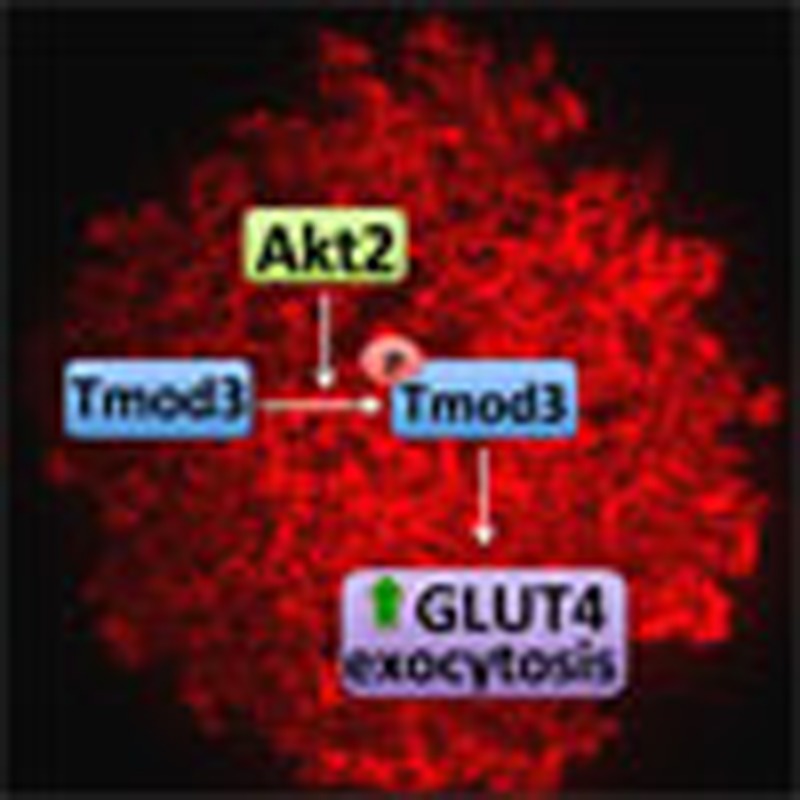

A major contributing factor to diabetes is the development of insulin resistance in peripheral metabolic organs in which insulin becomes progressively less effective in lowering plasma glucose level. A prominent insulin action is to promote glucose uptake into the muscle and fat cells by initiating PI3K/Akt signalling cascade to trigger the movement of a specialized pool of insulin-responsive glucose transporter, GLUT4, from intracellular compartments to the cell surface. Surface-exposed GLUT4 induced by the insulin signalling is responsible for lowering postprandial hyperglycaemia to normal glucose levels^1^,^2^,^3^. Impaired GLUT4 translocation is a major cellular manifestation of impaired insulin signalling pathway in patients with diabetes, in diet-induced obese animals and many transgenic animals associated with insulin resistance^4^,^5^.

Given the critical role of GLUT4 regulation in diabetes development, significant efforts have been devoted to understanding the cellular events and molecular control of insulin-stimulated GLUT4 translocation (ISGT). Insulin regulates multiple steps of GLUT4 translocation, including GSVs (GLUT4-storage vesicles) sorting, trafficking, tethering, docking and finally fusion^3^,^6^,^7^, through governing protein–protein interactions, protein–GSV association and, more importantly, post-translational modifications such as phosphorylation of different molecules involved in these steps. A key converging node of insulin action is protein kinase B or Akt2, whose essential role in ISGT and glucose transport has been demonstrated in numerous cell-based studies, in knockout mouse models and in diabetic patients carrying inactivating mutation of Akt2 (refs 8, 9, 10, 11). Akt2, which is recruited to the plasma membrane (PM) and activated via phosphorylation at Thr308 and Ser473 residues by PDK-1 and mTORC2, respectively, orchestrates the insulin actions by phosphorylating a number of downstream targets whose functional activities are necessary for many discrete steps of GSV exocytosis^2^,^12^.

To date, only several Akt2 substrates involved in ISGT have been identified. The most notable one, AS160, a Rab-GTPase-activating protein, is involved in GSVs retention at basal state and regulation of GSVs trafficking under insulin stimulation^13^. AS160-knockdown (KD) adipocytes exhibit increased basal level of cell surface GLUT4, and yet these cells remain sensitive to Akt-inhibitor treatment^14^, thus suggesting the presence of AS160-independent step(s) and/or additional Akt2 substrate(s) for optimal fusion of GLUT4 at the final stage of exocytosis. More recently, CDP138, a previously uncharacterized C_2_-domain containing protein in adipocytes was found to be an Akt2 substrate, which may imply the involvement of calcium binding or sensing in GLUT4 vesicle fusion^15^. Given the importance of Akt2 and its substrates in the regulation of glucose transport, identifying novel Akt2 substrates and defining their functions in GLUT4 translocation will provide new mechanistic insights into dysregulation of glucose homeostasis in diseased states.

Regarding actions beneath the PM, insulin promotes GLUT4 insertion by increasing the rate of vesicle fusion possibly by regulating cortical actin filament organization at exocytotic sites^7^,^16^,^17^,^18^,^19^. Several actin filament inhibitors have been shown to impair GLUT4 translocation and glucose uptake in response to insulin stimulation^20^,^21^. The inhibition of GLUT4 translocation by Latrunculin B (refs 21, 22) indicates the critical requirement of the formation of new actin filaments. Furthermore, studies of GSV dynamics demonstrate that the actin filaments are most likely involved in the tethering of vesicles close to the PM^19^,^23^. The myosin motor Myo1c (refs 24, 25) was recently shown to mediate insulin-induced tethering of GSVs to submembranous actin filaments^23^.

Although insulin-induced remodelling of actin filaments into cortical mesh is essential for GLUT4 translocation^26^,^27^, the direct link between Akt2 and actin reorganization is largely unclear. In this study, we report that Tropomodulin 3 (Tmod3), a pointed-end actin-capping protein, is an insulin-stimulated Akt2 substrate necessary for ISGT in adipocytes. KD of Tmod3 inhibits insulin-stimulated GLUT4 PM insertion and glucose uptake. In Tmod3-depleted cells, re-expression of phosphorylation-mimetic mutant S71D potentiates and phosphorylation-defective mutant S71A mutant impairs the insulin-stimulated GLUT4 exocytosis and glucose uptake. By examining actin dynamics in living adipocytes under total internal reflection fluorescence microscopy (TIRFM), we demonstrate a critical role of Tmod3 and its phosphorylation in the process of insulin-induced actin remodelling, an essential step for GSV fusion with the PM. Altering Tmod3-G-actin binding through Akt2-induced Tmod3 phosphorylation may, at least in part, mediates insulin-dependent actin remodelling. Moreover, we identify Tm5NM1 as a cognate tropomyosin (Tm) partner for Tmod3 in adipocytes and their interaction is necessary for ISGT and glucose uptake. Taken together, our study provides a mechanistic link between Akt2 signalling and the formation of a specific population of actin filaments in the process of GSV–PM fusion.

## Results

### Tmod3 is phosphorylated by Akt2 on insulin stimulation

**Figure 1:**
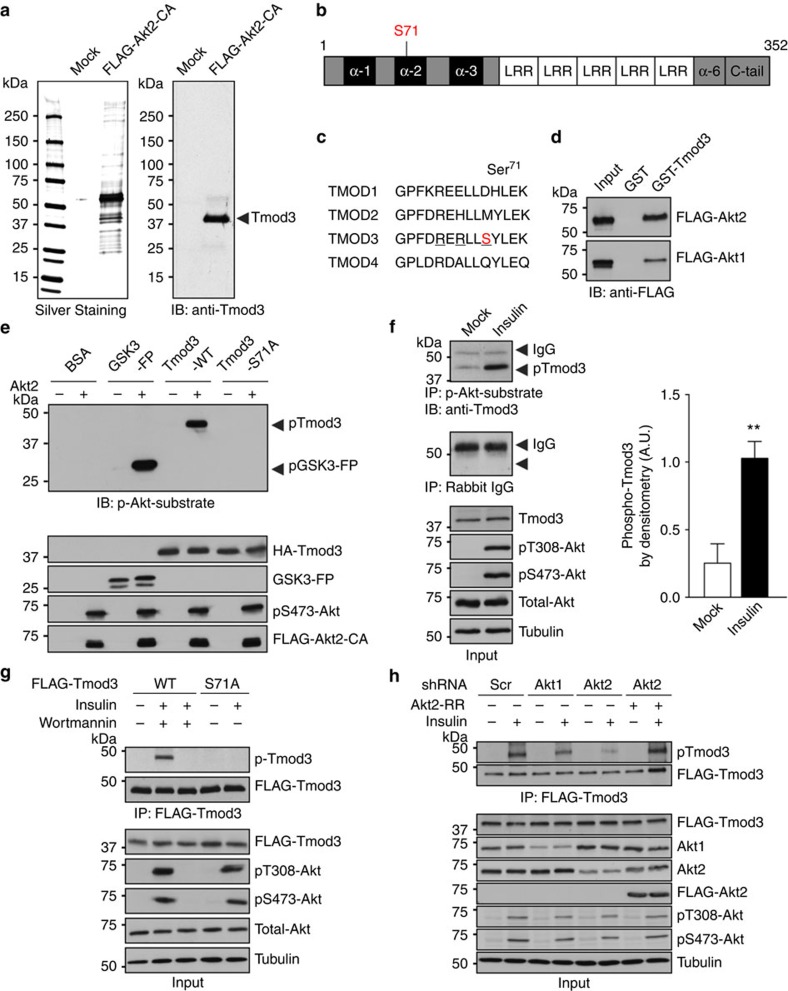
Tmod3 is a novel Akt2 substrate. (**a**) Silver-stained gel showing affinity-purified FLAG-Akt2-CA (myr-FLAG-Akt2) and its bound proteins from 3T3-L1 adipocytes, left panel; immunoblot showing anti-Tmod3 on pull down with anti-FLAG M2 beads, right panel. Arrowhead indicates Tmod3. (**b**) Schematic drawing showing the domain structures of Tmod3. (**c**) The Akt consensus sequence is present only in Tmod3. (**d**) Akt–Tmod3 interaction as detected by GST-pull-down assay. GST-fusion protein beads were incubated with lysates of HEK293T expressing FLAG-Akt1 or Akt2. (**e**) *In vitro* kinase assays showing direct phosphorylation of Tmod3 at Ser71 by Akt2. GSK3 was used as a positive control (GSK3-FP). (**f**) Insulin-stimulated phosphorylation of endogenous Tmod3 in 3T3-L1 adipocytes. 3T3-L1 differentiated adipocytes were subjected to insulin stimulation for 20 min. Anti-PAS was used for the immunoprecipitation of phosphorylated Akt substrates from cell lysates of adipocytes followed by immunoblotting with Tmod3 Ab, left panel; quantification of phospho-Tmod3 bands by densitometry, mean±s.d. (*n*=4; unpaired Student’s *t*-test). **P<0.01 versus Mock treatment, right panel. (**g**) Insulin-stimulated Tmod3 phosphorylation in 3T3-L1 adipocytes stably expressing FLAG-Tmod3 (WT or S71A). Anti-FLAG M2 beads were used to pull down Tmod3 from cell lysates followed by immunoblotting with anti-PAS and re-blotting with anti-FLAG Ab. (**h**) Tmod3 phosphorylation by Akt2, but not Akt1, in 3T3-L1 adipocytes. Phosphorylation of Tmod3 was diminished in the absence of Akt2, but restored when an shRNA-resistant human Akt2 was introduced into Akt2-deficient cells. Data are representative of at least two to three independent experiments except **a**. See also Supplementary Fig. 1.

To search for novel Akt substrates involved in ISGT, we performed a proteomic screening in 3T3-L1 adipocytes expressing FLAG-tagged constitutively active form of Akt2 (myristoylated human Akt2, FLAG-Akt2-CA). Spontaneous phosphorylation and activation of Akt2 at the PM is sufficient to stimulate GLUT4 translocation and glucose uptake in 3T3-L1 adipocytes to an extent similar to insulin stimulation^28^,^29^. Using this approach, we identified a 40-kDa protein named Tmod3 as a potential Akt2-interacting protein (Fig. 1a). Tmod3, a ubiquitously expressed pointed-end actin-capping protein^30^,^31^, was present in 3T3-L1 pre-adipocytes and differentiated adipocytes (Supplementary Fig. 1a,b). Sequence analysis revealed a single Akt consensus phosphorylation motif [RERLLS*, Ser71] located within the second α-helix region (α-2, amino-acid position 67 to 77) near the amino (N) terminus of Tmod3 (Fig. 1b). The phosphorylation motif is unique to Tmod3 and is not present in other tropomodulin isoforms (Fig. 1c). Together these results suggest Tmod3 as a substrate for Akt2.

We first confirmed the direct interaction between Tmod3 and Akt2 by GST-pull-down assay (Fig. 1d) and mapped the carboxyl (C)-terminal leucine-rich repeats domains of Tmod3 as necessary for its interaction with Akt2 (Supplementary Fig. 1c). We then performed *in vitro* kinase assays to determine whether Tmod3 is a direct substrate of Akt2, and found that wild-type (WT) Tmod3, but not S71A mutant, was phosphorylated, indicating that S71 is a phosphorylation site by Akt2 (Fig. 1e and Supplementary Fig. 1d). Moreover, when incubated with Akt2, robust incorporation of ^32^P into Tmod3 was detected by autoradiography (Supplementary Fig. 1e). To examine whether Tmod3 is phosphorylated *in vivo*, we co-expressed Tmod3 in HEK293T cells with either constitutively active (FLAG-Akt2-CA) or kinase-dead Akt2 (FLAG-Akt2-DN, R274H mutation)^9^ and performed co-immunoprecipitation studies using anti-HA or anti-FLAG affinity beads or anti-PAS (phospho-Akt substrate) accordingly. WT Tmod3 was phosphorylated in the presence of active Akt2, whereas S71A mutant was not (Supplementary Fig. 1f). Furthermore, mass spectrometry (MS) analysis of Tmod3 purified from HEK293T cells co-expressing Tmod3 and active Akt2 showed that Akt2 specifically phosphorylated Tmod3 at Ser71, but not other residues (Supplementary Fig. 1g). These findings support that Tmod3 is directly phosphorylated at S71 by Akt2 both *in vitro* and *in vivo*.

Furthermore, endogenous Tmod3 was markedly phosphorylated in adipocytes treated with insulin, and the phosphorylation was largely abolished when the cells were pretreated with PI3K or Akt inhibitors (Fig. 1f and Supplementary Fig. 1h). Similar results were obtained in adipocytes stably expressing FLAG-Tmod3, and in white adipose tissues from insulin-injected C57BL/6 mice, further confirming that phosphorylation of Tmod3 is dependent on insulin signalling (Fig. 1g and Supplementary Fig. 1i). As both Akt1 and Akt2 isoforms are expressed in 3T3-L1 adipocytes, we then determined which Akt is the primary kinase for Tmod3 phosphorylation. KD of Akt2 in adipocytes significantly reduced the phosphorylation of Tmod3 (Fig. 1h). In contrast, KD of Akt1 showed only mild effect on Tmod3 phosphorylation. Furthermore, the expression of short hairpin RNA (shRNA)-resistant human Akt2 restored Tmod3 phosphorylation in Akt2-deficient cells on insulin stimulation. Taken together, these data indicate that Tmod3 is primarily an Akt2 substrate and its phosphorylation is regulated by insulin signalling pathway.

### Tmod3 is required for ISGT and glucose uptake

Tropomodulin proteins contribute to the geometry of the membrane cytoskeleton by blocking the elongation and depolymerization of the pointed end of actin filaments^30^. In particular, Tmod3 localizes preferentially to the F-actin-rich structures at the cell periphery, including lamellipodia and ruffles^32^. Consistently, we found that Tmod3 at the cell periphery was associated with cortical F-actin at the sites of insulin-induced membrane ruffles and co-localized with GLUT4 (Supplementary Fig. 2). Given the importance of Akt signalling and cortical actin remodelling in regulating GSV translocation, it is conceivable that Tmod3 may be involved in the regulation of ISGT and glucose uptake through cortical actin remodelling.

**Figure 2:**
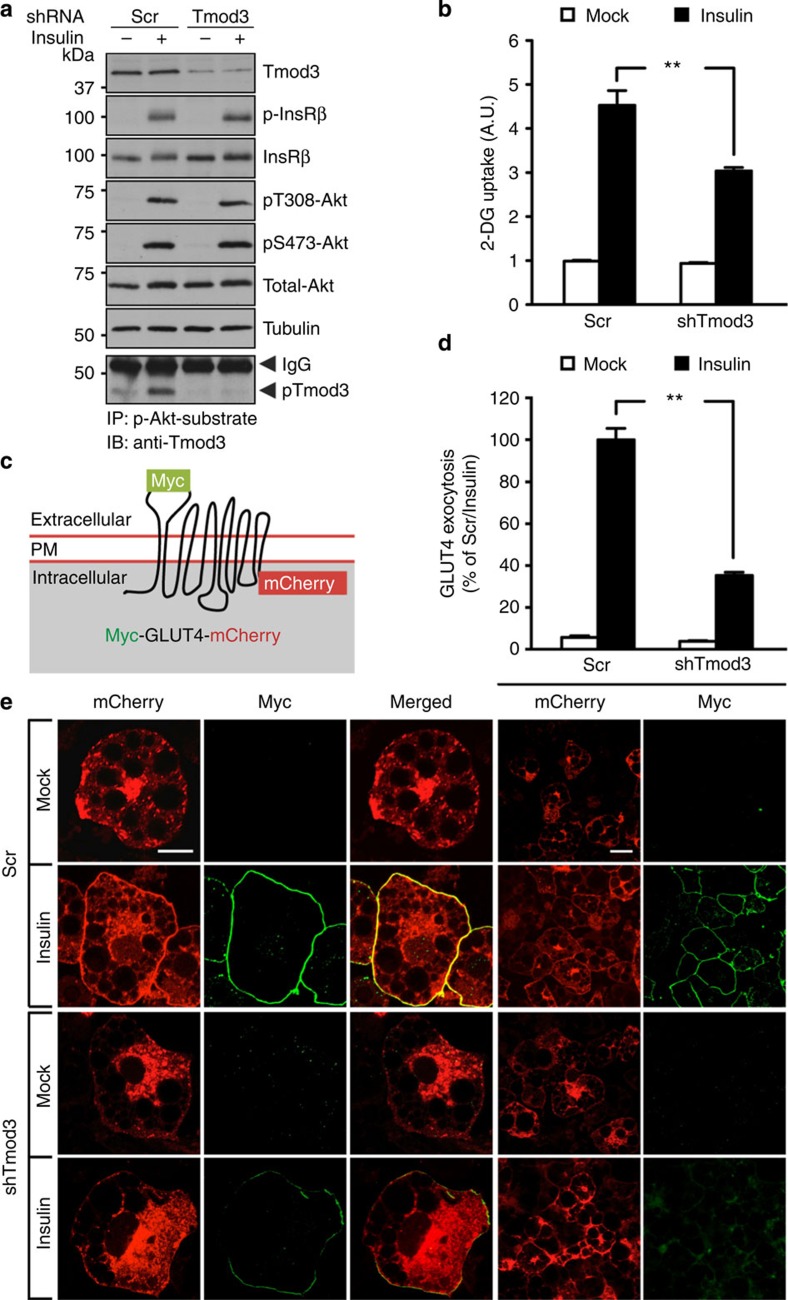
Tmod3 functions downstream of Akt2 signalling and is required for ISGT and glucose uptake. (**a**) Normal proximal insulin signalling in Tmod3-KD 3T3-L1 adipocytes. After 2-h serum starvation, the cells received mock or insulin treatment for 20 min. The specificity of anti-PAS antibody to pull-down phosphorylated Tmod3 was confirmed in Tmod3-KD cells, bottom panel. (**b**) Impaired glucose uptake in Tmod3-KD 3T3-L1 adipocytes. After 3-h serum starvation, the cells received mock or insulin treatment for 20 min for measurement of 2-DG uptake. Data are expressed as mean±s.e.m. (*n*=4; analysis of variance (ANOVA) with Dunnett’s multiple comparison test). **P<0.01 versus Scr Insulin groups. (**c**) GLUT4 fusion protein for detection of GLUT4 translocation and PM surface exposure. (**d**,**e**) Impaired insulin-stimulated GLUT4 surface exposure in Tmod3-KD 3T3-L1 adipocytes. The cells received mock or insulin treatment for 20 min after 2-h serum starvation. The ratio of surface to total GLUT4 was quantified by detecting surface GLUT4 through anti-Myc fluorescence immunolabelling and total GLUT4 through mCherry fluorescence in non-permeabilized cells. Data in each group were normalized and expressed as a percentage of insulin-treated control cells. Data presented are representative confocal microscopic images and means±s.e.m. of about 100 cells in each group from three independent experiments (ANOVA with Dunnett’s multiple comparison test). **P<0.01 versus Scr Insulin groups. Scale bars in **e**, 20 μm.

To investigate the function of Tmod3, we generated stable 3T3-L1 cells with Tmod3 KD. Compared with control cells, KD of Tmod3 had no effect on proximal insulin signalling in 3T3-L1 adipocytes, suggesting that Tmod3 functions downstream of Akt signalling (Fig. 2a). We next examined the involvement of Tmod3 in insulin-stimulated glucose uptake, and found that Tmod3-KD cells displayed ~40% reduction in glucose uptake when compared with control cells (Fig. 2b). Since insulin-induced glucose uptake is primarily mediated by GLUT4 in adipocytes, we tested whether GLUT4 exocytotic pathway was altered due to the loss of Tmod3. We generated a lentiviral construct to express a Myc-GLUT4-mCherry fusion protein in which the Myc epitope was inserted in the first exofacial loop of GLUT4 N terminus, and mCherry fused at the C terminus (Fig. 2c), thus allowing detection of GLUT4 on PM insertion (by Myc in non-permeabilized condition) and of total GLUT4 content (by mCherry), similar to two other reported constructs^33^,^34^. In control cells, insulin induced significant GLUT4 presence at the cell surface, while silencing of Tmod3 reduced the GLUT4 presence (Fig. 2d,e). These results indicate that Tmod3 is necessary for ISGT and glucose uptake in adipocytes.

### Tmod3 is required for GLUT4 insertion into the PM

**Figure 3:**
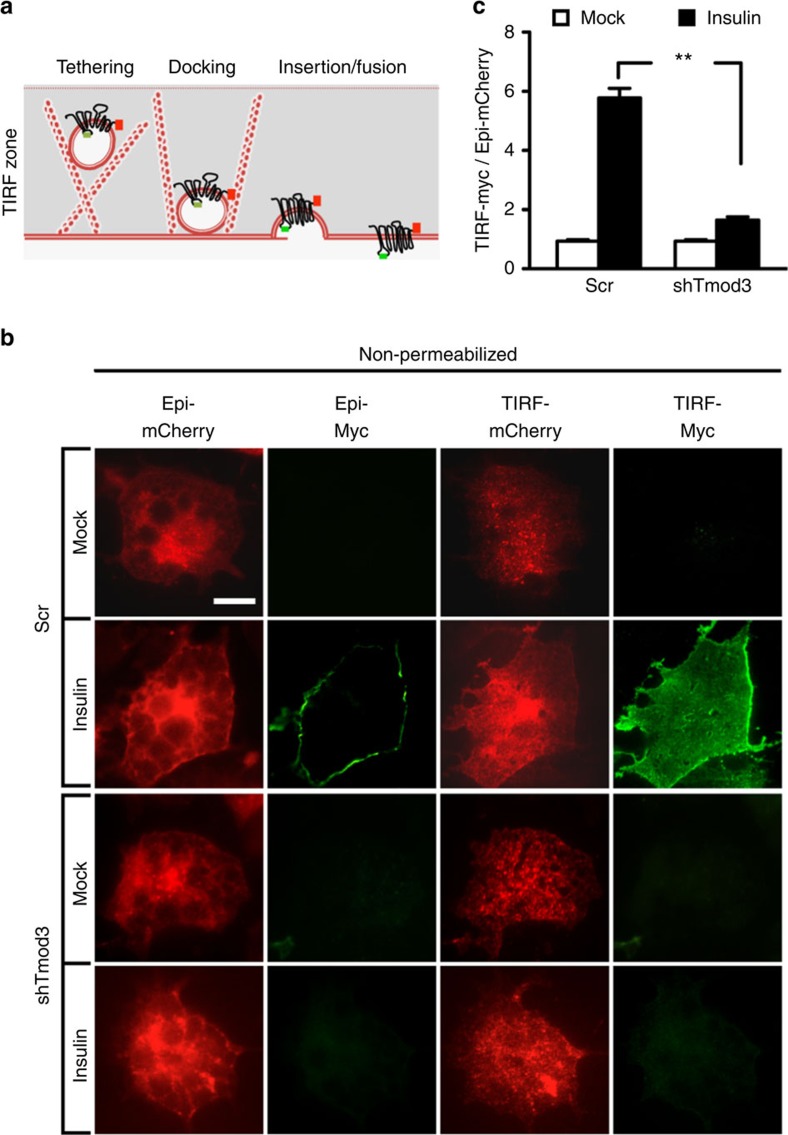
Tmod3 is required for insulin-stimulated GLUT4 vesicle fusion but not trafficking of GSVs to the periphery. (**a**) Schematic drawing illustrating events observed under total internal reflection fluorescence imaging zone (TIRF zone). (**b**,**c**) Impaired GSV fusion with PM in Tmod3-KD 3T3-L1 adipocytes. Insulin-induced GLUT4 membrane insertion was examined by using TIRF microscopy in non-permeabilized cells. GLUT4 insertion is shown as the ratio of cell surface TIRF-Myc signal to total Epi-mCherry. Data presented are representative microscopic images and means±s.e.m. of about 50 cells in each group from three independent experiments (analysis of variance with Dunnett’s multiple comparison test). **P<0.01 versus Scr Insulin groups. Scale bars in **b**, 20 μm.

When examining the confocal images on the distribution of Myc-GLUT4-mCherry in Tmod3-KD adipocytes, we noticed that a substantial amount of GLUT4 accumulated at the cell periphery as revealed by mCherry fluorescent signals, and yet the cells showed weak green fluorescent signals, suggesting that silencing of Tmod3 results in impairment of GLUT4 membrane insertion without affecting the mobilization of GSVs from the intracellular storage compartment (Fig. 2e). To further test this notion, we performed TIRFM to examine the distribution of Myc-GLUT4-mCherry within ~200 nm from the PM under non-permeabilized condition in fixed cells (Fig. 3a), and used the ratio of Myc fluorescence under TIRFM to mCherry fluorescence under epifluorescence microscopy to indicate the extent of GLUT4 membrane insertion. As expected, significant ISGT was observed in control cells as indicated by intense green signal at the cell periphery under both epifluorescence and TIRFM (Fig. 3b,c). In contrast, only weak Myc signals were detected in non-permeabilized Tmod3-KD adipocytes under TIRFM, indicating reduced PM insertion of GLUT4 (Fig. 3b,c). The observed intense Myc signals at the cell periphery in permeabilized Tmod3-KD cells confirmed that GLUT4 trafficking from intracellular storage compartment was not affected (Supplementary Fig. 3a). To confirm that our approach could discern the GLUT4 trafficking defects, we imaged adipocytes with Akt2 KD, which was reported to show impaired GSV mobilization from intracellular storage compartment^5^,^15^. Akt2-KD cells exhibited a significant reduction of GLUT4 at the cell periphery as revealed by concomitant loss of both red and green fluorescent signals (Supplementary Fig. 3b). We also imaged adipocytes that were treated with Latrunculin B, which was shown to disrupt cortical actin and impair GLUT4 fusion^22^,^27^. As expected, Latrunculin B treatment led to significantly reduced GLUT4 membrane insertion with no apparent decrease in GLUT4 trafficking to the cell periphery (Supplementary Fig. 3c,d). Taken together, these findings suggest that Tmod3 is specifically required for GLUT4 membrane insertion, possibly the GSV–PM fusion step, but not its mobilization or translocation to the PM.

### Tmod3 phosphorylation potentiates ISGT

**Figure 4:**
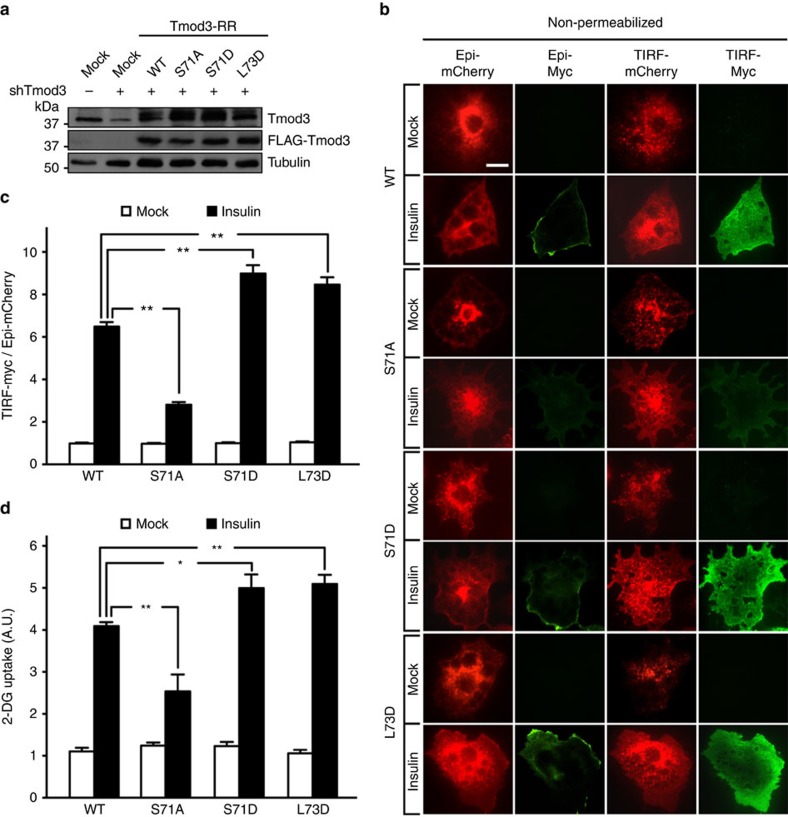
Phosphorylation of Tmod3 promotes insulin-stimulated GLUT4 exocytosis and glucose uptake in adipocytes. (**a**) Western blots showing the expression levels of endogenous Tmod3 and overexpressed FLAG-Tmod3 variants in adipocytes. (**b**,**c**) Phosphomimetic Tmod3-S71D potentiates, while phosphodefective Tmod3-S71A inhibits GSVs fusion with PM. Insulin-stimulated GLUT4 insertion was examined by TIRF-based Myc-GLUT4-mCherry assay under non-permeabilized conditions. The ratio of cell surface TIRF-Myc signal to total Epi-mCherry signal is presented as mean±s.e.m. of about 100 cells in each group from three independent experiments (analysis of variance (ANOVA) with Dunnett’s multiple comparison test). **P<0.01 versus WT insulin groups. Representative TIRF images are shown in **b**. Scale bar, 20 μm. (**d**) Effects of Tmod3 variants on glucose uptake in adipocytes. After 3-h serum starvation, cells received mock or insulin treatment for 20 min for measurements of 2-DG uptake (*n*=4; ANOVA with Dunnett’s multiple comparison test). *P<0.05, **P<0.01 versus WT insulin groups.

Having shown that Tmod3 is required for GLUT4 membrane insertion, we then examined whether S71 phosphorylation played any role in Tmod3 function, particularly on insulin-stimulated GSV exocytosis and glucose uptake. We first generated lentiviral constructs to express shRNA-resistant WT or variants of Tmod3 in stable Tmod3-KD adipocytes (Fig. 4a). Under insulin stimulation, Tmod3-KD cells expressing phosphodefective Tmod3-S71A showed significantly lower surface presence of GLUT4 when compared with the cells expressing WT Tmod3 (Fig. 4b,c). On the other hand, cells expressing phosphomimetic S71D showed further enhancement of insulin-induced GLUT4 exocytosis (Fig. 4b,c). It is worth noting that mutation at L73 (L73D), which disrupts the second α-helix of Tmod3 and decreases both its actin monomer-binding and -nucleating activities^35^, elicited a similar effect as that seen for S71D mutation (Fig. 4b,c). Consistent with altered GLUT4 membrane insertion, glucose uptake was reduced in cells expressing Tmod3-S71A, and increased in those expressing Tmod3-S71D or Tmod3-L73D (Fig. 4d). Together these results support that Tmod3 phosphorylation positively regulates insulin-stimulated GSV fusion with the PM, the final step for ISGT where actin remodelling occurs.

### Tmod3 regulates insulin-induced actin remodelling

**Figure 5:**
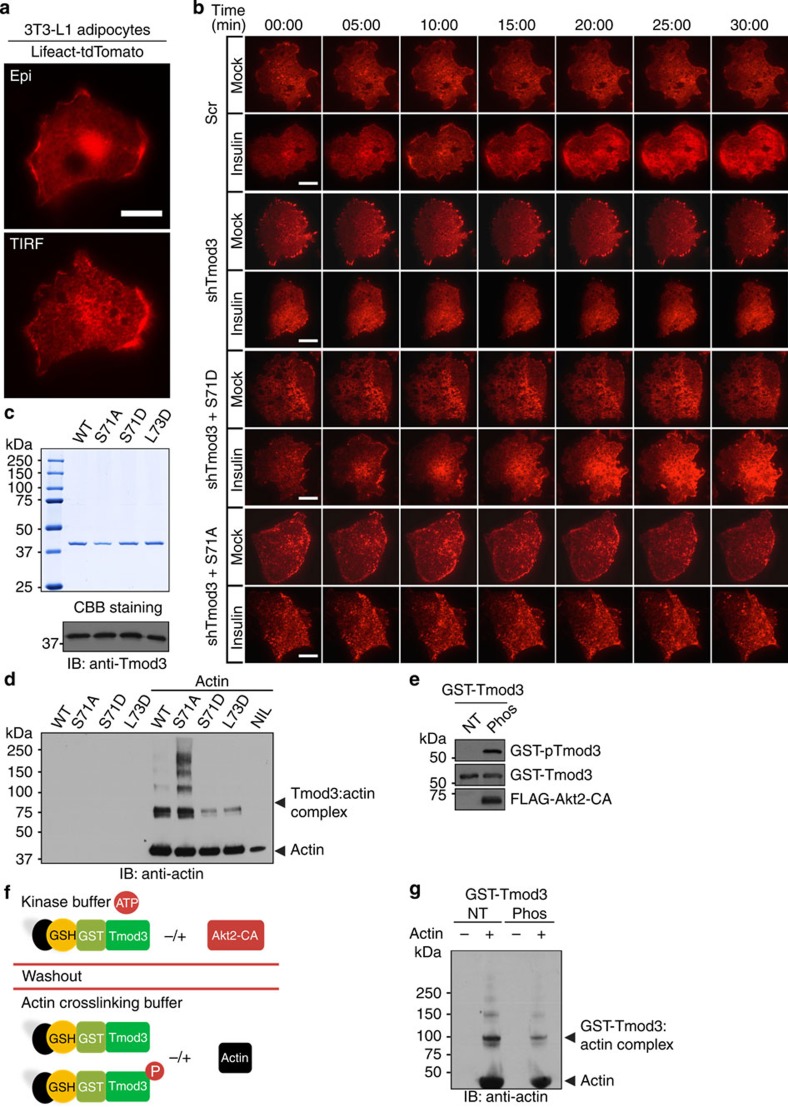
Tmod3 phosphorylation regulates its binding to monomeric G-actin and insulin-induced actin remodelling. (**a**) TIRF image of F-actin structures in 3T3-L1 adipocytes expressing Lifeact-tdTomato. (**b**) Representative time-lapse images of actin remodelling in selected cells expressing Lifeact-tdTomato on shRNA KD and re-expression of Tmod3 mutants. Original movies for these images are in Supplementary Movies 1a,b and 2a–f. See Supplementary Fig. 4 for time course analysis and details of measurement in Methods. Scale bars in **b**, 20 μm. (**c**) Recombinant Tmod3 proteins were visualized by Coomassie Blue staining and confirmed by western blot using anti-Tmod3 Ab. (**d**) Crosslinking experiments using recombinant Tmod3 proteins and G-actin show reduced interaction between phosphomimetic Tmod3 and G-actin, but enhanced binding between phosphodefective Tmod3 and G-actin. Crosslinking complexes were detected by anti-actin. (**e**,**f**) Purified GST-Tmod3 WT proteins (**e**) were subjected to *in vitro* Akt2 kinase assay in the absence or presence of purified constitutively active Akt2 (NT, not treated with Akt2 and therefore not phosphorylated or Phos: treated with Akt2 and phosphorylated), followed by *in vitro* actin-crosslinking assay (**g**), as described in the schematic diagram (**f**). (**g**) Phosphorylated Tmod3 group showing significant reduction of Tmod3–actin complexes as detected by anti-actin.

Insulin has been reported to promote actin polymerization and to increase the rate of actin remodelling in adipocytes^22^,^27^. A previous study using live-cell TIRF microscopy on 3T3-L1 adipocytes expressing actin-eGFP revealed that insulin treatment stimulated an enrichment of cortical actin in membrane ruffles and increased the rate of ventral actin polymerization^22^. To investigate whether phosphorylation of Tmod3 could influence the activities of insulin-induced cortical actin remodelling, we generated a 3T3-L1 cell line expressing Lifeact-tdTomato, a fluorescent F-actin marker^36^ to visualize actin remodelling in live cells (Fig. 5a). Consistent with previous reports^22^,^27^, when compared with mock treatment, insulin stimulation led to significantly increased cortical actin structures in the immediate vicinity of the PM as well as increased ventral polymerized actin as shown by increased Lifeact-tdTomato fluorescence under TIRFM (Fig. 5b; Supplementary Movie 1a,b). Although both peripheral and ventral actin dynamics were implicated in promoting vesicle fusion events, it remained unclear the relative significance of these pools in contributing to insulin-induced actin remodelling. We first analysed the time course of Lifeact fluorescence specifically on the periphery area of the cells using a quantitative method, which could discern the events of mere membrane movement from the events of actin remodelling, as described in the Methods. Insulin-stimulated enrichment of cortical actin usually results in fluorescence oscillations with a sustained increase in intensity level, which is distinguishable from moderate oscillations returning to baseline under mock condition (Supplementary Fig. 4a). We also performed time course analysis specifically on the ventral regions excluding the cell periphery to assess the influence of ventral actin polymerization (Supplementary Fig. 4). As expected, cells treated with Latrunculin B showed progressive loss of actin filaments and accumulation of actin ‘dots’ (polymer clusters) in the intracellular region as well as the cell periphery. Subsequent treatment with insulin failed to effect reorganization of actin filaments in Latrunculin B-treated cells (Supplementary Fig. 5a; Supplementary Movie 1c,d). Depletion of Tmod3 in adipocytes attenuated the activities of insulin-dependent actin remodelling, resulting in modest buildup of cortical actin and decreased the rate of ventral actin polymerization (Fig. 5b and Supplementary Fig. 4b; Supplementary Movie 2a,b). We then examined Tmod3 mutants on insulin-induced actin remodelling under TIRFM. Re-expression of shRNA-resistant Tmod3-S71D, but not S71A, in Tmod3-KD cells led to increased membrane ruffles and pronounced thickening of both cortical and ventral actin under insulin-stimulated condition (Fig. 5b and Supplementary Fig. 4; Supplementary Movie 2c–f). Moreover, we observed that actin remodelling in Akt2-KD cells was impaired (Supplementary Fig. 5b,c; Supplementary Movie 2g,h). Taken together, these results suggest a critical role for Tmod3 in actin remodelling, and mechanistically, Tmod3 phosphorylation may serve as a regulatory node of Akt2 signalling to fine tune the process of actin rearrangements.

A common feature for actin-binding proteins is their ability to bind monomeric G-actin^35^,^37^. Tmod3 has several functional domains: an N-terminal domain comprising three α-helices, followed by five leucine-rich repeat domains, and a C-terminal domain capable of binding to F-actin filaments directly. Within the N-terminal domain, α-helix-1 and -3 are necessary for interacting with a Tm partner, while α-helix-2 is important for G-actin-binding and Tm-dependent actin capping^35^,^37^,^38^. We tested the ability of Tmod3 mutants in G-actin binding by *in vitro* actin-crosslinking assay (Fig. 5c). Consistent with previous reports^30^,^35^,^37^, WT Tmod3, when mixed with G-actin, readily formed complexes with apparent molecular mass of 80–160 kDa, as detected by anti-actin antibody (Fig. 5d). Moreover, Tmod3-S71A mutant showed dramatically increased complexes with G-actin, while phosphomimetic Tmod3-S71D significantly decreased the formation of actin–Tmod3 complexes, similar to Tmod3-L73D (Fig. 5d). We also performed *in vitro* Akt2 kinase assay to phosphorylate the GST-Tmod3 WT proteins and compared crosslinking activities between non-phosphorylated and phosphorylated forms of GST-Tmod3 WT (Fig. 5e,f). Similar to Tmod3-S71D, phosphorylated GST-Tmod3 showed reduced complex formation with G-actin when compared with equal amount of non-phosphorylated GST-Tmod3 (Fig. 5g), further supporting that Tmod3 phosphorylation by Akt2 regulates its binding to monomeric G-actin and actin assembly. Taken together, these results suggest that modifying Tmod3 in G-actin-binding activity through Akt2-mediated phosphorylation at S71 may, at least in part, contribute to insulin-dependent actin remodelling.

### Tmod3–Tm5NM1 interaction is necessary for ISGT

Tropomodulin proteins bind specifically to the pointed end of the actin filament and control actin polymerization by regulating the stability, lengths and architecture of actin filaments. However, the binding affinity of tropomodulin to actin filaments is modest at the basal state, which can be increased dramatically in the presence of Tm^39^. The interaction between Tmod and Tm is important in regulating actin pointed-end capping activity of all Tropomodulins. Moreover, the pairing of Tmod-Tm is selective, with different combinations of Tmod and Tm isoforms having distinct binding preferences and affinities, depending on the expression patterns, cell type and developmental stages^30^. To identify the cognate Tm-binding partner for Tmod3 in adipocytes, we performed immunoprecipitation studies in 3T3-L1 adipocytes stably expressing FLAG-Tmod3. Although Tm1, Tm3, Tm4 and Tm5NM1 were expressed in 3T3-L1 adipocytes as assessed by western blotting, only Tm5NM1 was found to interact specifically with Tmod3 (Supplementary Fig. 6).

**Figure 6:**
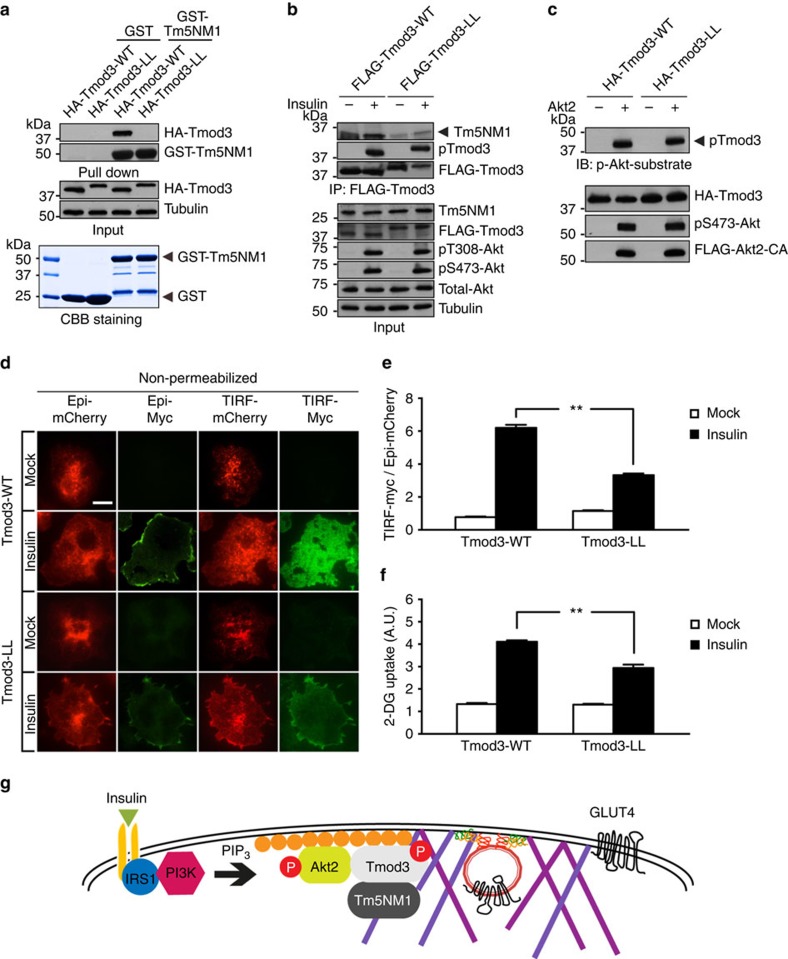
Tmod3–Tm5NM1 interaction is essential for ISGT and glucose uptake. (**a**) Loss of Tm5NM1 binding with Tmod3-L29G/L134D (Tmod3-LL) mutant by *in vitro* GST-pull-down assay. GST-Tm5NM1 beads were incubated with lysates of HEK293T expressing HA-Tmod3-WT or HA-Tmod3-LL. (**b**) Impaired binding between endogenous Tm5NM1 and Tmod3-LL mutant in adipocytes. (**c**) Tmod3-LL mutant remains sensitive to Akt2-mediated phosphorylation *in vitro*. (**d**,**e**) Insulin-stimulated GSV exocytosis by TIRF-based Myc-GLUT4-mCherry assay shows significantly reduced GLUT4 surface exposure in Tmod3-LL cells. The ratio of cell surface TIRF-Myc signal to total Epi-mCherry signal is presented as mean±s.e.m. of about 100 cells in each group from three independent experiments (analysis of variance (ANOVA) with Dunnett’s multiple comparison test). **P<0.01 versus WT insulin groups. Representative microscopic images are shown in **d**. Scale bar, 20 μm. (**f**) Ectopic expression of Tmod3-LL mutant inhibits insulin-stimulated glucose uptake in adipocytes. Cells received mock or insulin treatment for 20 min after 3-h serum starvation for 2-DG uptake measurement (*n*=4; ANOVA with Dunnett’s multiple comparison test). **P<0.01 versus WT insulin groups. (**g**) Schematic model for Tmod3 as a molecular link between Akt2 activation and actin remodelling in insulin-stimulated GLUT4 fusion with PM. See Discussion for details.

To address the functional relevance of Tmod3–Tm5NM1 interaction, we generated a mutant Tmod3 defective in this interaction. Since both the first and third α-helices of Tmod3 are necessary for its Tm interaction, we generated a Tmod3 mutant harbouring both L29G and L134D mutations, equivalent to L27E/I131D in Tmod1 (ref. 40), and showed that Tmod3-L29G/L134D (Tmod3-LL) completely lost binding to Tm5NM1 (Fig. 6a). We also expressed the double mutant Tmod3-LL in adipocytes and found that it showed significantly reduced interaction with endogenous Tm5NM1 (Fig. 6b). Furthermore, Tmod3-LL retained insulin- and Akt2-induced phosphorylation (Fig. 6c). We then examined whether the Tmod3–Tm5NM1 interaction played any role in ISGT and glucose uptake. When compared with WT Tmod3, the expression of Tmod3-LL mutant in adipocytes inhibited ISGT and resulted in impaired glucose uptake (Fig. 6d–f). Collectively, these data support a role of Tmod3–Tm5NM1 interaction in ISGT and glucose uptake.

## Discussion

ISGT serves as a primary mechanism of glucose disposal by skeletal muscle and adipose tissues^3^. Biochemical and genetic studies have established the critical importance of Akt, especially Akt2 in mediating the ISGT process^1^,^4^,^10^. As such, the identification of Akt substrates that are involved in regulating the GSV exocytosis and understanding the cellular and molecular mechanisms of their actions in glucose transport will enable us to pinpoint the major nodes of molecular pathways underlying this process, as defects in this process are responsible for impaired glucose uptake in insulin resistance and diabetes. Although the mechanisms that regulate membrane fusion of GLUT4 are not completely understood, it has become clear that cortical actin remodelling plays a pivotal role^18^,^19^,^20^,^21^,^27^,^41^,^42^, in coordination with the membrane fusion machinery and its regulators, including SNARE proteins, small GTPases and Sec-1/Munc18 proteins, to bridge and eventually merge the two lipid bilayers between GSVs and the PM, thus allowing cell surface exposure of GLUT4 proteins and the ensuing glucose uptake^1^. In relation to the importance of cortical actin dynamics to the pathogenesis of insulin resistance and diabetes, previous reports have shown that PM PIP_2_ and cortical F-actin structure are reduced in both 3T3-L1 adipocytes and L6 myotubes with chronic insulin or high-glucose treatment, leading to impaired ISGT and glucose transport^19^,^27^,^41^. Furthermore, high-fat diet-fed insulin-resistant mice show reduced F-actin in skeletal muscle compared with normal chow-fed mice^43^.

Insulin-induced cortical actin remodelling, in particular dynamic cortical actin rearrangement, but not the static actin barrier, is necessary for ISGT in adipocytes and muscle cells^19^,^26^,^27^,^42^. Regulation of insulin-dependent actin remodelling may vary depending on tissue or cell types. For example, a previous study^44^ reported that a PI3K-independent pathway, involving the activation of TC10, is required for F-actin assembly to support the insulin-stimulated GLUT4 exocytosis in adipocytes, along with several other studies that propose actin-associated proteins such as N-WASP and the myosin motor Myo1c to function downstream of the wortmannin-insensitive pathway^24^,^45^. However, numerous studies on muscle cells and muscle tissue^19^,^42^,^46^,^47^,^48^,^49^ suggest that cortical actin remodelling is largely PI3K-dependent and requires the activation of the small GTPase Rac1 (refs 42, 47). Furthermore, the Arp2/3 complex-mediated actin branching and cofilin-facilitated actin severing have been proposed to function downstream of Rac1 in regulating actin polymerization/depolymerization cycles, which are essential for GLUT4 insertion and exposure at the cellular surface^46^. Notably, the overexpression of a dominant negative Rac1 inhibited insulin-dependent actin modelling and decreased GLUT4 translocation in muscle cells^42^,^50^, but had no effect on basal or insulin-stimulated glucose uptake in adipocytes^51^,^52^. Hence, it is possible that the muscle cells and adipocytes use different mechanisms in regulating actin remodelling during ISGT. Moreover, it remains to be clarified whether PI3K-independent pathway may necessarily preclude the direct involvement of Akt kinase in cortical actin reorganization especially in adipocytes. In earlier studies on muscle cells, neither Akt dominant negative mutant nor Akt inhibitor prevented insulin-induced Rac activation or actin remodelling, and Rac KD had no effect on insulin-stimulated Akt phosphorylation^50^,^53^, suggesting that the two pathways function independently in supporting ISGT. However, more recent studies^54^,^55^ showed insulin-induced Rac1 activation in muscle cells could be suppressed by Akt2 KD and constitutively active forms of Akt2 and PI3K failed to induce GLUT4 translocation in gastrocnemius muscle fibres of muscle-specific *rac1* KO mice, thereby indicating that Rac1 may be a critical downstream effector of Akt2 in mouse skeletal muscle instead. Inconsistency of these observations between different studies may be due to specific experimental conditions and strategies, and hence warrants further investigations and clarifications.

In our study, KD of Tmod3 in adipocytes has no influence on proximal insulin signalling, as insulin-stimulated Akt activation remains intact. KD of Akt2, by contrast, significantly decreases insulin-mediated phosphorylation of Tmod3. The identification of Tmod3 as an Akt2 substrate may imply a role for Akt kinase in cortical actin reorganization, at least partially through modulating Tmod3 activity via phosphorylation. Indeed, by visualizing actin dynamics in live cells with TIRF microscopy, we demonstrate that the depletion of Tmod3 or Akt2 by shRNA inhibits the enrichment of cortical actin and decreases the rate of ventral actin polymerization beneath the PM in adipocytes. It remains to be determined which pool of actin is primarily involved in contributing to insulin-induced vesicle fusion events. These two types of actin network are distinct from each other in terms of structural morphology and length: cortical actin structures are condensed and relatively longer, whereas ventral actin structures are shorter in length, yet significantly different from stress fibres as seen in typical fibroblasts. We are not aware of any comprehensive study in delineating the specific roles of these two distinct actin networks in adipocytes or in differentiating their molecular compositions that drive the remodelling process. This is clearly an important but complex problem that requires future studies. To this end, we have performed time course measurement of the TIRF intensities of Lifeact-tdTomato at the cell periphery and the ventral regions excluding the cell periphery, respectively. Our observation in adipocytes supports that insulin induces a temporal burst of cortical actin wave and also increases the overall rate of ventral actin polymerization.

Given that actin remodelling is a dynamic process, disrupting the actions of any actin-binding proteins through pharmacological or genetic approaches in the cycles of actin polymerization/depolymerization would presumably have an impact on the cytoskeletal rearrangements as a whole. As such, we cannot rule out that Tmod3 depletion may affect ISGT indirectly by causing a global change in the actin network. Another unresolved issue is the lack of concrete experimental evidence that directly demonstrates how actin structures participate in the vesicle fusion step under specific signalling pathways at the molecular level, even though numerous previous studies^19^,^27^,^42^,^46^ have shown impaired vesicle exocytosis when actin cytoskeleton is disrupted. At present, delineating specific roles of Tmod3 in actin remodelling at vesicle fusion sites remains a challenging task. This is an important question and warrants future detailed investigations. Nonetheless, there is no significant difference between Tmod3-KD cells and control cells in the basal level of glucose uptake or the basal level of surface GLUT4. Moreover, a key observation of this study is that the re-expression of phosphomimetic Tmod3 mutant in Tmod3-KD cells rescues the defect in insulin-dependent actin remodelling, paralleling a potentiation in ISGT and glucose uptake. It is plausible that phosphorylation of Tmod3 provides a mechanism for Akt2 to fine tune the insulin-dependent actin remodelling in preparation of subsequent docking and fusion by the tethered GSVs. Interestingly, both S71D and L73D mutant cells do not display increased basal level of glucose uptake or increased basal gain in surface GLUT4 when compared with WT cells. As the inactivation of AS160 and subsequent mobilization of GSVs by Rab GTPases^13^,^56^ from intracellular storage compartments precede the action of Tmod3 on actin behaviour at the cortical area, these findings suggest that the phosphorylation of Tmod3 alone is necessary but not sufficient to orchestrate the entire process of insulin-dependent GLUT4 translocation and exocytosis. Indeed, only GLUT4 surface exposure, possibly the GSV–PM fusion step, but not GSVs arrival at the TIRF zone, is affected in Tmod3-KD cells. On the other hand, AS160-KD cells, as shown in the previous studies by others^14^,^56^,^57^, exhibit increased basal gain in surface GLUT4 but still show impairment of GSV–PM fusion in insulin-stimulated condition, indicating that additional steps are required for optimal GSV translocation and exocytosis. It is tempting to suggest that insulin-induced actin enrichment beneath the PM as one of these regulatory steps to coordinate precise positioning of GSVs and their access and interaction with the SNARE fusion machinery.

At the molecular level, Tmod3 interacts with monomeric G-actin via its second α-helix^35^,^37^,^58^. Interestingly, the Tm-dependent actin-capping activity mediated by Tmod3 also requires intact second α-helix^35^. Mutagenesis of L73 to D, which disrupts the second α-helix of Tmod3, decreases both its actin monomer-binding and -nucleating activities. In our study, the phosphorylation of Tmod3 at S71 decreased actin monomer binding and potentiated the insulin-stimulated GLUT4 exocytosis. It remains to be determined whether Tmod3 could function as an actin monomer-sequestering protein in the cellular context besides its well-known role in actin capping at the pointed end. One plausible mechanism is that release of G-actin from Tmod3 on phosphorylation may provide an immediate supply of actin monomer to facilitate the rapid buildup of cortical actin at the exocytosis sites to allow GSV–PM fusion. Indeed, Tmod3 phosphorylation affected its ability to bind G-actin (Fig. 5d,g). However, it is technically challenging to assess the monomer-sequestering activity *in vivo* at the cellular level. Moreover, whether the actin monomer sequestration by Tmod3 is independent of its Tm-dependent F-actin-capping activity requires further investigations. A recent study^58^ showed that Tmod1 and Tmod3 similarly cap actin filaments with diverse Tm and actin isoforms, but only Tmod3 sequesters β- and γ(cyto)-actin monomers, suggesting that Tmod3-mediated monomer-sequestering activity may have a role in regulating actin remodelling or turnover in the absence of Tm. Interestingly, insulin is known to induce dephosphorylation and activation of cofilin in various cell types^46^,^59^,^60^. Activated cofilin severs actin filaments at the pointed end, the same site where Tmod3 acts. Cofilin KD via siRNA in muscle cells readily increased F-actin aggregates at the basal state and inhibited ISGT, which could not be rescued by the expression of inactive cofilin-S3E-GFP^46^. It is possible that Tmod3 may complement cofilin, under the regulation of insulin signalling, in generating ample supply of actin monomer to induce a spatial and temporal burst in cortical actin remodelling.

The identification of Tmod3 as a downstream target for Akt phosphorylation also provides a mechanism by which insulin specifies the assembly of actin filaments with specific functional properties. Tropomodulins, encoded by four genes in mammals, primarily function by binding to the pointed end of actin filaments to slow down the turnover of actin filaments^30^. Studies using genetically manipulated mice and cells have shown that the level of tropomodulin can control actin filament length^38^,^61^. There are two broad classes of actin filaments, one with just the two-stranded actin polymer, and the other comprising a co-polymer of both actin and Tm^62^,^63^. There are more than 40 Tm isoforms, however, cytoskeletal actin filaments are largely, if not entirely, composed of homopolymers of specific Tm dimers^64^,^65^. Besides providing a structural homogeneity to the filament, the Tm homodimer bound to the actin filament determines the functional capacity of the filament through its specific interactions with actin-binding proteins and myosin motors^62^,^63^,^66^. In the case of adipocytes, Tmod3 shows a clear preference for binding to Tm5NM1/2-decorated actin filaments. Such binding will significantly promote the pointed-end capping activities of Tmod3, leading to increased length and stability of cortical actin filaments^30^,^64^. In addition, the selective pairing of Tmod3 with Tm5NM1 may be important to recruit a specific actin-based myosin motor that drives the vesicle to the exocytotic site^62^,^66^. Tm5NM1 is known to recruit the myosin motor MyoIIA to actin filaments^62^ and MyoIIA has recently been shown to be required for ISGT in adipocytes^67^. It is possible that the generation of Tmod3/Tm5NM1 filaments recruits MyoIIA and results in reorganization of these filaments in the cortex of the cell. Our study identifies Tm5NM1 as the cognate Tm partner for Tmod3 and establishes the importance of the Tmod3–Tm5NM1 interaction for efficient ISGT and glucose uptake.

In summary, we propose that insulin signalling regulates GSV fusion with PM through cortical actin rearrangement, which is at least in part mediated by Akt2-induced phosphorylation of Tmod3. We report identification and demonstration of Tmod3 as an Akt2-interacting partner and substrate in adipocytes. Tmod3 plays a functional role in ISGT and glucose uptake, particularly at the step before vesicle fusion. Under insulin stimulation, Akt2 mediates phosphorylation of Tmod3 at cell periphery. Phosphorylated Tmod3 and its Tm partner, Tm5NM1, elicit rearrangement of a specific pool of actin filament to promote GSV fusion and GLUT4 surface exposure (Fig. 6g). Our study highlights the role and mechanism of Akt2-mediated phosphorylation of Tmod3 in insulin-stimulated cortical actin remodelling and GLUT4 insertion into the PM.

## Methods

### Reagents

All the plasmids and antibodies are listed in Supplementary Tables 1 and 2. All chemicals were purchased from Sigma unless otherwise stated.

### Mouse work

Mice (C57BL/6, male, 12 weeks old) used in this study were maintained in A*STAR Biological Resource Centre animal facility. All animal experiments were approved by the A*STAR Institutional Animal Care and Use Committee (#110683).

### Cell culture

3T3-L1 fibroblasts (ATCC) were cultured and differentiated into adipocytes by established protocols^68^. In brief, 3T3-L1 fibroblasts were cultured in high-glucose DMEM (Invitrogen, GIBCO) supplemented with 10% newborn calf serum at 37 °C and 5% CO_2_. Two days after confluence, cells were washed with PBS twice and switched into differentiation medium containing 10% fetal bovine serum (FBS), 1 μM insulin, 0.5 mM 3-isobutyl-1-methylxanthine and 1 μM dexamethasone for 2 days. The differentiation medium was then replaced with media containing 10% FBS and 167 nM insulin for another 2 days. Cells were then maintained in DMEM with 10% FBS. Adipocytes were used for experiments at day 10 post differentiation. HEK293T (ATCC) and CHO-IR cells (from Jae Bum Kim) were cultured in DMEM with 10% FBS.

### Lentivirus packaging and infection

Lentiviruses were produced by co-transfecting HEK293T cells with lentiviral expression and packaging plasmids using the calcium phosphate transfection method^68^. Viral supernatant was collected 24 h after transfection, centrifuged at 1,000 *g* for 5 min and filtered with 0.45 μm filter. The viruses were further concentrated by ultracentrifugation using SW41 swinging rotor in Beckman Coulter Optima-L-100 XP at 100,000 g for 2 h at 20 °C. 3T3-L1 adipocytes at day 4 of differentiation were infected with concentrated viruses in the presence of 4 μg ml^−1^ of polybrene. Culture media containing viruses were replaced with fresh DMEM media 48 h after infection and infected adipocytes were only subjected to experiments at day 10 post differentiation.

### *In vitro* protein kinase assay

*In vitro* kinase assays were performed on purified HA-tagged Tmod3 WT and mutant proteins. In brief, 1 μg of HA-Tmod3 and 50 ng of FLAG-AKT2-CA were incubated in 50 μl of volume reaction in kinase buffer (25 mM Tris-Cl, pH 7.5, 5 mM β-Glycerophosphate, 2 mM DTT (dithiothreitol), 0.1 mM Na_3_VO_4_, 10 mM MgCl_2_) containing 0.2 mM ATP. The kinase reaction was incubated at 30 °C for 30 min and boiled in SDS sample buffer and separated by SDS-polyacrylamide gel electrophoresis (SDS–PAGE). GSK-3β fusion protein (GSK-3β FP, Cell Signaling) was used as a positive control. Phosphorylation signals were detected using anti-PAS Ab. As for the radioactive method, the sample mixtures were incubated in the kinase buffer with 10 μCi [^32^P] γ-ATP (PerkinElmer, SG) for 30 min at 30 °C. Samples were analysed by SDS–PAGE and autoradiography.

### *In vitro* GST-pull-down assay

GST-fusion proteins were produced and purified from bacteria lysates with glutathione resin (GE Healthcare) as described in the Protein Purification section. The glutathione beads coupled to GST-fusion proteins were equilibrated with TNET buffer and then incubated with appropriate amount of cell lysates for indicated time at 4 °C. Pull-down samples were collected and washed four times with TNET buffer. Samples were subjected to SDS–PAGE and western blot analysis.

### *In vitro* actin-crosslinking assay

Purified non-muscle actin from human platelet was purchased from Cytoskeleton, Inc. Each protein sample was centrifuged at 300,000 *g* at 4 °C for 2 h to remove aggregates, and protein concentrations were determined by spectrophotometry. Crosslinking of G-actin and Tmod3 was performed according to established protocol with slight modifications^35^. In brief, G-actin and Tmod3 proteins were exchanged into a buffer containing 5 mM HEPES, pH 7.5, 0.1 mM CaCl_2_, 0.2 mM ATP and 0.2 mM DTT using protein desalting spin columns (Pierce). G-actin and Tmod3 were mixed at a 1:1 molar ratio at concentrations between 10 and 20 μM for 20 min at room temperature. Crosslinking stock solutions of EDC and sulfo-NHS (Pierce) were freshly prepared and added to the reaction at a final concentration of 1 mM for additional 45 min at room temperature. Reactions were terminated by adding 2 × SDS sample buffer followed by SDS–PAGE. Assays using GST-Tmod3 proteins were performed similarly except that the proteins were bound to glutathione beads.

### Non-radioactive 2-deoxyglucose uptake assay

Non-radioactive 2-deoxyglucose uptake assays were conducted^69^. Infected adipocytes were seeded on 12-well plates and serum starved for 3 h. Cells were washed three times with warm KRPH (Krebs Ringer Phosphate Hepes) buffer (1.2 mM KH_2_PO_4_, 1.2 mM MgSO_4_, 1.3 mM CaCl_2_, 118 mM NaCl, 5 mM KCl, 30 mM HEPES, pH 7.5). Cells were incubated with 1 ml KRPH buffer containing 0.5% bovine serum albumin and 2 mM sodium pyruvate at 37 °C and stimulated with or without 100 nM insulin for 20 min. Glucose uptake was initiated by the addition of 1 mM 2-deoxyglucose for 20 min at 37 °C. Assays were terminated by washing cells with cool PBS three times, the cells per well were lysed with 0.5 ml of 10 mM Tris-HCl, pH 8.0 supplemented with 0.5% Triton-X-100 and subjected to heat treatment at 80 °C for 15 min. Lysates were then centrifuged at 15,000*g* for 20 min at 4 °C. Diluted samples (40 × ) were used for measurement of 2-deoxyglucose uptake using the non-radioactive 2-deoxyglucose uptake ELISA kit (Cosmo Bio Co., Japan) according to manufacturer’s instructions. In brief, the endogenous glucose-6-phosphate was first eliminated by oxidation to 6-phosphogluconate (6PG) with NAD^+^ and a low concentration of glucose-6-phosphate dehydrogenase. The endogenous NAD(P)H as well as NADH produced in the first step were removed with HCl. Levels of 2DG6P were then quantitated by measuring the amount of NADPH produced during 2DG6P oxidation (with NADP^+^ and a high concentration of glucose-6-phosphate dehydrogenase) in a photometric recycling amplification/detection system. Non-specific 2-deoxyglucose uptake was measured in the presence of 20 μM cytochalasin B and subtracted from all groups.

### Fluorescence microscopy

For co-localization studies, adipocytes plated on 1% gelatin-coated coverslips were serum starved before insulin treatment. Cells were fixed with 4% paraformaldehyde, washed with PBS and blocked with PBS containing with 2% FBS and 0.02% sodium azide for at least 1 h. Cells were then permeabilized with 0.1% saponin in PBS and stained with relevant antibodies followed by Alexa Fluor secondary antibodies accordingly. After washing, the coverslips were mounted with DAKO mounting medium (DAKO). Optical sections of samples were taken through sequential scans at relevant wavelengths using a Nikon A1R-A1 confocal laser microscope system with a × 100 NA/1.40 CFI Plan APO VC oil-immersion objective. TIRFM setup was based on Nikon Eclipse-Ti inverted microscope with two EMCCD cameras (1,002 × 1,002 pixels, 8 × 8 μm, 14-bit, Andor iXon^EM^+885; Andor Technologies) capable of capturing two channels (TIRF laser 488 nm; TIRF laser 561 nm) simultaneously. Both TIRF and epifluorescence images were captured using a × 100 NA/1.49 APO TIRF oil-immersion objective. Immersion oil (ND=1.515, Nikon) was used to bridge the optical contact between the objective and the coverslip. The penetration depth of the evanescent field is estimated to be ~200 nm. Images were acquired with no binning; at 27 MHz, the readout rate with average exposure times vary between 50 and 100 ms. Cells were reseeded to grow in 35-mm glass-bottom dishes (MatTEK) for 2 days before imaging. Cells were serum starved for 2 h and incubated in imaging buffer at 37 °C with insulin treatment. Cells were kept in a Tokai Hit temperature controlled incubation box at 37 °C supplemented with 5% CO_2_.

### Myc-GLUT4-mCherry translocation

To examine the effect of KD of Tmod3 or expression of Tmod3 mutants on GLUT4 translocation, cells were transduced with lentivirus encoding shRNA against Tmod3 or Tmod3 mutants accordingly. In non-permeabilized condition, cells were incubated with primary mouse anti-Myc Ab (9E10) followed by Alexa Fluor 488-conjugated anti-mouse secondary Ab. Mounted samples were subjected to confocal imaging. Nikon Element software was used for quantitative measurements of GLUT4 translocation. In brief, the entire ventral surface of individual cells expressing Myc-GLUT4-mCherry was selected for measurement of fluorescence intensity of both Myc- and mCherry after removal of background fluorescence. The ratio of surface to total GLUT4 was quantified by detecting surface GLUT4 through anti-Myc fluorescence immunolabelling and total GLUT4 through mCherry fluorescence in non-permeabilized cells. Data in each group were normalized and expressed as a percentage of insulin-treated control cells. TIRF/epifluorescence analysis for fixed cells was performed^15^,^70^. Under non-permeabilized conditions, the ratio of TIRF-Myc/Epi-mCherry was acquired by dividing the fluorescence intensity of TIRF-Myc signal for each cell by that of the Epifluorescence GLUT4-mCherry. Data in each group were averaged and the ratio of TIRF-myc/Epi-mCherry in Scr control group was set as 1 for group comparison.

### TIRFM-based actin remodelling and analysis

3T3-L1 adipocytes expressing Lifeact-tdTomato were serum starved for 2 h and imaged using TIRFM. Insulin was added at zero time point and TIRF images were taken over 30 min with an interval of 15 s. Two independent ways of measurement were used to analyse the actin remodelling. (1) Measurement of TIRF-Lifeact-tdTomato on the cell periphery: as for this analysis, we focused particularly on the insulin-stimulated buildup or enrichment of cortical actin at the cell periphery. To quantify, multiple boxes of region of interest (L × W: 8 × 5, 40 μm^2^ per region of interest (ROI)) spanning across the boundary of cell periphery were used to measure the fluorescence intensities of RAW images over time. Each ROI was manually selected to include a portion of the cell periphery and partial background to take into account the membrane reorganization in the time course. After removal of the background fluorescence, TIRF intensities of all ROIs measured over time were normalized to the intensity measured at zero time point, averaged and plotted against the time to indicate the time course of actin remodelling. This method selectively estimated the extent of cortical actin rearrangements without considering the changes in actin behaviour, if any, occurring in intracellular ventral region. Events of thickening of cortical actin could be distinguished from the events of membrane remodelling: fluorescence oscillations with a sustained increase in intensity level resulted from thickening of cortical actin, whereas oscillations returning to baseline usually reflected mere membrane movement. (2) Measurement of TIRF-Lifeact-tdTomato on the ventral regions away from the cell edge: ventral regions of individual cells excluding the regions of the cell periphery were manually selected in Nikon Element software and subjected to time course analysis as described in (1). This method selectively assessed the extent of ventral actin polymerization over time without taking into account the changes in peripheral actin structures and membrane ruffling activity.

### Protein purification

(A) Enrichment of FLAG-Akt2-CA and its associated complexes for mass spectrometry study: 3T3-L1 adipocytes at day 4 were infected with lentivirus carrying myr-FLAG-human Akt2 and harvested at day 10. Cells were lysed in TNET buffer (50 mM Tris-Cl, pH 7.5, 150 mM NaCl, 1 mM EDTA, 10 mM NaF, 1% Triton-X-100) containing 1 mM Na_3_VO_4_, 1 mM PMSF (phenylmethyl sulphonyl fluoride) and protease inhibitor cocktail tablet (Roche). Cleared supernatant was used for immunoprecipitation with anti-FLAG M2 beads. Bound complexes were washed extensively with TNET buffer and eluted with 3 × FLAG peptide in 20 mM NH_4_HCO_3_, pH 8.3. The eluate of the samples was subjected to mass spectrometry analysis. (B) Preparation of HA-/FLAG-tagged proteins from HEK293T cells: HA- and FLAG-tagged proteins were expressed in HEK293T cells and purified using EZview Red Anti-HA Affinity Gel or anti-FLAG M2 beads, respectively. Proteins were subsequently eluted with HA-peptide or 3 × FLAG peptide in TBS buffer (50 mM Tris-Cl, pH 7.5, 150 mM NaCl). Protein concentrations were measured using Bradford assay. (C) Recombinant GST-fusion protein purification: Full-length mouse Tmod3 and mutants were inserted in-frame in the linker region of pGEX-KG vector so as to encode a fusion protein with GST on the N terminus. GST-fusion Tmod3 and mutants were expressed in BL21 *Escherichia coli* and induced for protein production with 1 mM isopropyl-β-D-thiogalactoside for 2 h when the OD_600_ of bacteria growth reached ~1.00. Bacteria were then harvested and proteins were purified using standard protocol. In brief, bacteria pellets were suspended in Buffer A (20 mM Tris-Cl, pH 8.0, 0.1 M NaCl, 1 mM DTT) containing 1 mM PMSF and protease inhibitor cocktail tablet (Roche) and incubated with 0.25 mg ml^−1^ lysozyme on ice for 30 min. The bacterial lysate was sonicated on ice using an ultrasonic processor Vibra-Cell VCX130 (Sonics & Materials, Inc.) with eight cycles of a 30-s pulse followed by a 10-s break at 40% amplitude output for 4 min. The lysate was treated with 0.5% Triton-X-100 and rocked at 4 °C for 15 min. The lysate was cleared by centrifugation at 14,000 r.p.m. at 4 °C for 30 min. The pre-equilibrated glutathione-Sepharose 4B beads (GE Healthcare) in Buffer A with 0.1% Triton-X-100 was incubated with clear supernatant for 1 h at 4 °C. GST-fusion proteins were bound to glutathione beads and eluted by cleaving with thrombin (GE Healthcare) in TTB buffer (20 mM Tris-Cl, pH 8.0, 150 mM NaCl, 2.5 mM CaCl_2_). Thrombin was then removed using Benzamidine-Sepharose beads (GE Healthcare). Thrombin-free eluted proteins were further concentrated using appropriate centrifugal filter units (Amicon Ultra, Millipore). Protein concentrations were measured before the assays.

### Mass spectrometric analysis

(A) SDS–PAGE gel based ID: FLAG-tagged constitutively active form of Akt2 and its associated protein complexes were pulled down using anti-FLAG M2 beads (Sigma-Aldrich) and released with glycine-HCl, pH 3.0. The eluate of the samples was lyophilized at −80 °C and reconstituted in 30 μl of SDS sample buffer, resolved in 4–15% TGX gradient SDS–PAGE and detected with silver staining (Supplementary Fig. 7). Visible bands were excised from the gel, subjected to reduction with 25 mM DTT at 56 °C and alkylation with 55 mM iodoacetamide in the dark at room temperature, followed by in-gel digestion with trypsin (Promega, mass spectrometry grade), 10 ng μl^−1^ in 25 mM ammonium bicarbonate buffer, pH 8.0 for 16 h at 37 °C. Peptides were extracted and spotted onto MADLI target plate with HCCA as matrix. MALDI-TOF-TOF MS/MS were carried out at Bruker Ultraflex III TOF-TOF with supports of software including FlexControl 3.0, FlexAnalysis 3.0 and Biotools 3.2. Processed spectra peak lists were submitted to in-house Mascot server for database search. (B) Shotgun LC-Q-TOF MS/MS based ID: the above cell lysate was pulled down and directly eluted in the 25 mM ammonium bicarbonate buffer with 3 × FLAG peptide. After freeze drying, the protein mixtures were denatured in 20 μM of ammonium bicarbonate buffer containing 0.1% SDS and 25 mM DTT at 95 °C for 5 min and digested with 2 μg trypsin (Promega) for 16 h at 37 °C. Peptide mixtures were acidified by adjusting the concentration of 0.1% trifluoroacetic acid, and then injected to Waters SYNAPT Q-TOF MSMS analysis with the mobile phase B (acetonitrile+0.1% FA) gradient from 5% to 60% within 60 min in nanoACQUITY UPLC BEH130 C18 column 1.7 μm, 75 μm × 150 mm. The Masslynx generated pkl files were submitted to Mascot server for protein ID. (C) Detection of spectra of Phospho-Ser71 of Tmod3: FLAG-tagged Tmod3 proteins were purified from HEK293T transiently co-expressing FLAG-Tmod3 and constitutively active form of Akt2 (myr-HA-Akt2) using anti-FLAG M2 beads. Proteins were eluted in 25 mM ammonium bicarbonate buffer with 3 × FLAG peptide, concentrated by Vivaspin2 30 kDa MWCO PES ultrafiltration spin column (Sartorius Stedium Biotech, Germany) to 50 μl, treated with 10 mM DTT for 10 min at 90 °C and alkylated in 20 mM iodoacetamide for 30 min in the dark at room temperature. Samples were then incubated with 1 μg trypsin for 16 h at 37 °C. Peptide mixtures were extracted with C18 ZipTip pipette tips (Millipore) after acidification with trifluoroacetic acid and drying and reconstituted with 0.1% Formic acid, followed by HPLC CHIP with 150 mm × 75 μm ReproSil-Pur C18-AQ 5 μm separation on Agilent 1,260 Infinity Nanoflow LC and analysed on 6520 Q-TOF MS/MS. Spectra were processed with MassHunter workstation software (Agilent) and exported in mzData format for Mascot database search. Carbamidomethyl cysteine was set as fixed modification, whereas oxidized methionine, *N*-acetyl amino acids and phospho-STY were searched as variable modifications. Peptide mass tolerance was set to ±100 p.p.m. and fragment mass tolerance was set to ±0.2 Da. A maximum of three missed cleavages was allowed.

### Gene expression analysis by RT-PCR

Total RNA from 3T3-L1 adipocytes or epiWAT was extracted using Trizol Reagent (Invitrogen)^68^. One μg of RNA after DNase I treatment was used for first-strand cDNA synthesis using Applied Biosystems TaqMan Reverse Transcription Reagents. A 10 × -diluted cDNA of samples was subjected to real-time PCR using SYBR Green dyes with appropriate primers. The relative expression of mRNA abundance was calculated using 2^−ΔΔ*C*t^ method.

### Statistical analysis

Data were expressed as means±s.e.m. unless otherwise stated. Statistical analyses were performed using unpaired Student’s *t*-test or one-way analysis of variance for group comparisons. The levels of statistical significance were presented as asterisks and defined in each figure legend together with the name of the statistical test accordingly.

## Author contributions

C.-Y.L. designed and performed most of the experiments, analysed the data and wrote the manuscript. X.B. performed the mass spectrometry analysis. J.B.K., D.W., P.W.G., W. Hong edited the manuscript. W. Han supervised all aspects of this work and wrote the paper. All authors were involved in research design and discussion and approved the paper.

## Additional information

**How to cite this article**: Lim, C.-Y. *et al.* Tropomodulin3 is a novel Akt2 effector regulating insulin-stimulated GLUT4 exocytosis through cortical actin remodelling. *Nat. Commun.* 6:5951 doi: 10.1038/ncomms6951 (2015).

## Supplementary Material

Supplementary Information Supplementary Figures 1-7, Supplementary Tables 1-2 and Supplementary References

Supplementary Movie 1a | Time-lapse images of actin remodeling captured under TIRFM. Timelapse images were recorded for 30 min at an interval of 15 seconds Scr-Mock: Adipocyte expressing Lifeact-tdTomato was imaged using TIRFM at the basal state

Supplementary Movie 1b | Time-lapse images of actin remodeling captured under TIRFM. Timelapse images were recorded for 30 min at an interval of 15 seconds Scr-Ins: Adipocyte expressing Lifeact-tdTomato was imaged using TIRFM under 100nM insulin stimulation.

Supplementary Movie 1c | Time-lapse images of actin remodeling captured under TIRFM. Timelapse images were recorded for 30 min at an interval of 15 seconds Scr-Latrunculin B: Adipocyte expressing Lifeact-tdTomato was imaged using TIRFM under Latrunculin B treatment.

Supplementary Movie 1d | Time-lapse images of actin remodeling captured under TIRFM. Timelapse images were recorded for 30 min at an interval of 15 seconds Scr-Latrunculin B/Ins: after re-adjusting the focus, same cell in (c) was treated with insulin and imaged.

Supplementary Movie 2a | Time-lapse images of insulin-induced actin remodeling in cells uponknockdown and/or re-expression recorded under TIRFM. Time-lapse images were recorded for 30 min at an interval of 15 seconds shTmod3-Mock: Tmod3 KD cell was imaged using TIRFM at the basal state

Supplementary Movie 2b | Time-lapse images of insulin-induced actin remodeling in cells uponknockdown and/or re-expression recorded under TIRFM. Time-lapse images were recorded for 30 min at an interval of 15 seconds shTmod3-Ins: Tmod3 KD cell was imaged using TIRFM under 100nM insulin stimulation

Supplementary Movie 2c | Time-lapse images of insulin-induced actin remodeling in cells uponknockdown and/or re-expression recorded under TIRFM. Time-lapse images were recorded for 30 min at an interval of 15 seconds shTmod3+S71D-Mock: S71D mutant cell was imaged using TIRFM at the basal state

Supplementary Movie 2d | Time-lapse images of insulin-induced actin remodeling in cells uponknockdown and/or re-expression recorded under TIRFM. Time-lapse images were recorded for 30 min at an interval of 15 seconds shTmod3+S71D-Ins: S71D mutant cell was imaged using TIRFM under 100nM insulin stimulation

Supplementary Movie 2e | Time-lapse images of insulin-induced actin remodeling in cells uponknockdown and/or re-expression recorded under TIRFM. Time-lapse images were recorded for 30 min at an interval of 15 seconds shTmod3+S71A-Mock: S71A mutant cell was imaged using TIRFM at the basal state.

Supplementary Movie 2f | Time-lapse images of insulin-induced actin remodeling in cells uponknockdown and/or re-expression recorded under TIRFM. Time-lapse images were recorded for 30 min at an interval of 15 seconds shTmod3+S71A-Ins: S71A mutant cell was imaged using TIRFM under 100nM insulin stimulation.

Supplementary Movie 2g | Time-lapse images of insulin-induced actin remodeling in cells uponknockdown and/or re-expression recorded under TIRFM. Time-lapse images were recorded for 30 min at an interval of 15 seconds shAkt2-Mock: Akt2 KD cell was imaged using TIRFM at the basal state.

Supplementary Movie 2h | Time-lapse images of insulin-induced actin remodeling in cells uponknockdown and/or re-expression recorded under TIRFM. Time-lapse images were recorded for 30 min at an interval of 15 seconds shAkt2-Ins: Akt2 KD cell was imaged using TIRFM under 100nM insulin stimulation.

## References

[b1] Bogan J. S. Regulation of glucose transporter translocation in health and diabetes. Annu. Rev. Biochem. 81, 507–532 (2012). 22482906 10.1146/annurev-biochem-060109-094246

[b2] Rowland A. F., Fazakerley D. J. & James D. E. Mapping insulin/GLUT4 circuitry. Traffic 12, 672–681 (2011). 21401839 10.1111/j.1600-0854.2011.01178.x

[b3] Huang S. & Czech M. P. The GLUT4 glucose transporter. Cell Metab. 5, 237–252 (2007). 17403369 10.1016/j.cmet.2007.03.006

[b4] Stöckli J., Fazakerley D. J. & James D. E. GLUT4 exocytosis. J. Cell Sci. 124, 4147–4159 (2011). 22247191 10.1242/jcs.097063 PMC3258103

[b5] Leto D. & Saltiel A. R. Regulation of glucose transport by insulin: traffic control of GLUT4. Nat. Rev. Mol. Cell Biol. 13, 383–396 (2012). 22617471 10.1038/nrm3351

[b6] Foley K., Boguslavsky S. & Klip A. Endocytosis, recycling, and regulated exocytosis of glucose transporter 4. Biochemistry 50, 3048–3061 (2011). 21405107 10.1021/bi2000356

[b7] Hoffman N. J. & Elmendorf J. S. Signaling, cytoskeletal and membrane mechanisms regulating GLUT4 exocytosis. Trends Endocrinol. Metab. 22, 110–116 (2011). 21216617 10.1016/j.tem.2010.12.001 PMC3049829

[b8] Bae S. S., Cho H., Mu J. & Birnbaum M. J. Isoform-specific regulation of insulin-dependent glucose uptake by Akt/protein kinase B. J. Biol. Chem. 278, 49530–49536 (2003). 14522993 10.1074/jbc.M306782200

[b9] George S. *et al.* A family with severe insulin resistance and diabetes due to a mutation in AKT2. Science 304, 1325–1328 (2004). 15166380 10.1126/science.1096706 PMC2258004

[b10] Cho H. *et al.* Insulin resistance and a diabetes mellitus-like syndrome in mice lacking the protein kinase Akt2 (PKB beta). Science 292, 1728–1731 (2001). 11387480 10.1126/science.292.5522.1728

[b11] Garofalo R. S. *et al.* Severe diabetes, age-dependent loss of adipose tissue, and mild growth deficiency in mice lacking Akt2/PKB beta. J. Clin. Invest. 112, 197–208 (2003). 12843127 10.1172/JCI16885 PMC164287

[b12] Watson R. T. & Pessin J. E. GLUT4 translocation: the last 200 nanometers. Cell. Signal. 19, 2209–2217 (2007). 17629673 10.1016/j.cellsig.2007.06.003

[b13] Sano H. *et al.* Insulin-stimulated phosphorylation of a Rab GTPase-activating protein regulates GLUT4 translocation. J. Biol. Chem. 278, 14599–14602 (2003). 12637568 10.1074/jbc.C300063200

[b14] Brewer P. D., Romenskaia I., Kanow M. A. & Mastick C. C. Loss of AS160 Akt substrate causes Glut4 protein to accumulate in compartments that are primed for fusion in basal adipocytes. J. Biol. Chem. 286, 26287–26297 (2011). 21613213 10.1074/jbc.M111.253880 PMC3143591

[b15] Xie X. *et al.* C2 domain-containing phosphoprotein CDP138 regulates GLUT4 insertion into the plasma membrane. Cell Metab. 14, 378–389 (2011). 21907143 10.1016/j.cmet.2011.06.015 PMC3172579

[b16] Bai L. *et al.* Dissecting multiple steps of GLUT4 trafficking and identifying the sites of insulin action. Cell Metab. 5, 47–57 (2007). 17189206 10.1016/j.cmet.2006.11.013

[b17] Koumanov F., Jin B., Yang J. & Holman G. D. Insulin signaling meets vesicle traffic of GLUT4 at a plasma-membrane-activated fusion step. Cell Metab. 2, 179–189 (2005). 16154100 10.1016/j.cmet.2005.08.007

[b18] Brozinick J. T., Berkemeier B. A. & Elmendorf J. S. ‘Actin’g on GLUT4: membrane & cytoskeletal components of insulin action. Curr. Diabetes Rev. 3, 111–122 (2007). 18220662 10.2174/157339907780598199 PMC2396947

[b19] Tong P. *et al.* Insulin-induced cortical actin remodeling promotes GLUT4 insertion at muscle cell membrane ruffles. J. Clin. Invest. 108, 371–381 (2001). 11489930 10.1172/JCI12348 PMC209359

[b20] Tsakiridis T., Vranic M. & Klip A. Disassembly of the actin network inhibits insulin-dependent stimulation of glucose transport and prevents recruitment of glucose transporters to the plasma membrane. J. Biol. Chem. 269, 29934–29942 (1994). 7961991

[b21] Wang Q., Bilan P. J., Tsakiridis T., Hinek A. & Klip A. Actin filaments participate in the relocalization of phosphatidylinositol3-kinase to glucose transporter-containing compartments and in the stimulation of glucose uptake in 3T3-L1 adipocytes. Biochem. J. 331, 917–928 (1998). 9560323 10.1042/bj3310917 PMC1219436

[b22] Lopez J. A. *et al.* Identification of a distal GLUT4 trafficking event controlled by actin polymerization. Mol. Biol. Cell 20, 3918–3929 (2009). 19605560 10.1091/mbc.E09-03-0187 PMC2735490

[b23] Boguslavsky S. *et al.* Myo1c binding to submembrane actin mediates insulin-induced tethering of GLUT4 vesicles. Mol. Biol. Cell 23, 4065–4078 (2012). 22918957 10.1091/mbc.E12-04-0263 PMC3469521

[b24] Bose A. *et al.* Glucose transporter recycling in response to insulin is facilitated by myosin Myo1c. Nature 420, 821–824 (2002). 12490950 10.1038/nature01246

[b25] Yip M. F. *et al.* CaMKII-mediated phosphorylation of the myosin motor Myo1c is required for insulin-stimulated GLUT4 translocation in adipocytes. Cell Metab. 8, 384–398 (2008). 19046570 10.1016/j.cmet.2008.09.011

[b26] Kanzaki M. Insulin receptor signals regulating GLUT4 translocation and actin dynamics. Endocr. J. 53, 267–293 (2006). 16702775 10.1507/endocrj.kr-65

[b27] Kanzaki M. & Pessin J. E. Insulin-stimulated GLUT4 translocation in adipocytes is dependent upon cortical actin remodeling. J. Biol. Chem. 276, 42436–42444 (2001). 11546823 10.1074/jbc.M108297200

[b28] Ng Y., Ramm G., Lopez J. A. & James D. E. Rapid activation of Akt2 is sufficient to stimulate GLUT4 translocation in 3T3-L1 adipocytes. Cell Metab. 7, 348–356 (2008). 18396141 10.1016/j.cmet.2008.02.008

[b29] Kohn A. D., Summers S. A., Birnbaum M. J. & Roth R. A. Expression of a constitutively active Akt Ser/Thr kinase in 3T3-L1 adipocytes stimulates glucose uptake and glucose transporter 4 translocation. J. Biol. Chem. 271, 31372–31378 (1996). 8940145 10.1074/jbc.271.49.31372

[b30] Yamashiro S., Gokhin D. S., Kimura S., Nowak R. B. & Fowler V. M. Tropomodulins: Pointed-end capping proteins that regulate actin filament architecture in diverse cell types. Cytoskeleton 69, 337–370 (2012). 22488942 10.1002/cm.21031 PMC3444156

[b31] Cox P. R. & Zoghbi H. Y. Sequencing, expression analysis, and mapping of three unique human tropomodulin genes and their mouse orthologs. Genomics 63, 97–107 (2000). 10662549 10.1006/geno.1999.6061

[b32] Fischer R. S., Fritz-Six K. L. & Fowler V. M. Pointed-end capping by tropomodulin3 negatively regulates endothelial cell motility. J. Cell Biol. 161, 371–380 (2003). 12707310 10.1083/jcb.200209057 PMC2172920

[b33] Konstantopoulos N. & Molero-Navajas J. C. The measurement of GLUT4 translocation in 3T3-L1 adipocytes. Methods Mol. Biol. 560, 111–135 (2009). 19504248 10.1007/978-1-59745-448-3_10

[b34] Dawson K. Insulin-regulated trafficking of dual-labeled glucose transporter 4 in primary rat adipose cells. Biochem. Biophys. Res. Commun. 287, 445–454 (2001). 11554749 10.1006/bbrc.2001.5620

[b35] Yamashiro S., Speicher K. D., Speicher D. W. & Fowler V. M. Mammalian tropomodulins nucleate actin polymerization via their actin monomer binding and filament pointed end-capping activities. J. Biol. Chem. 285, 33265–33280 (2010). 20650902 10.1074/jbc.M110.144873 PMC2963411

[b36] Riedl J. *et al.* Lifeact: a versatile marker to visualize F-actin. Nat. Methods 5, 605–607 (2008). 18536722 10.1038/nmeth.1220 PMC2814344

[b37] Fischer R. S. *et al.* Tropomodulin 3 binds to actin monomers. J. Biol. Chem. 281, 36454–36465 (2006). 17012745 10.1074/jbc.M606315200

[b38] Gokhin D. S. *et al.* Tropomodulin isoforms regulate thin filament pointed-end capping and skeletal muscle physiology. J. Cell Biol. 189, 95–109 (2010). 20368620 10.1083/jcb.201001125 PMC2854367

[b39] Krieger I., Kostyukova A., Yamashita A., Nitanai Y. & Maéda Y. Crystal structure of the C-terminal half of tropomodulin and structural basis of actin filament pointed-end capping. Biophys. J. 83, 2716–2725 (2002). 12414704 10.1016/S0006-3495(02)75281-8 PMC1302356

[b40] Tsukada T. *et al.* Identification of residues within tropomodulin-1 responsible for its localization at the pointed ends of the actin filaments in cardiac myocytes. J. Biol. Chem. 286, 2194–2204 (2011). 21078668 10.1074/jbc.M110.186924 PMC3023515

[b41] McCarthy A. M., Spisak K. O., Brozinick J. T. & Elmendorf J. S. Loss of cortical actin filaments in insulin-resistant skeletal muscle cells impairs GLUT4 vesicle trafficking and glucose transport. Am. J. Physiol. Cell Physiol. 291, C860–C868 (2006). 16774991 10.1152/ajpcell.00107.2006 PMC2424226

[b42] Khayat Z. A., Tong P., Yaworsky K., Bloch R. J. & Klip A. Insulin-induced actin filament remodeling colocalizes actin with phosphatidylinositol 3-kinase and GLUT4 in L6 myotubes. J. Cell Sci. 113, 279–290 (2000). 10633079 10.1242/jcs.113.2.279

[b43] Habegger K. M. *et al.* Fat-induced membrane cholesterol accrual provokes cortical filamentous actin destabilisation and glucose transport dysfunction in skeletal muscle. Diabetologia 55, 457–467 (2012). 22002007 10.1007/s00125-011-2334-y PMC3245823

[b44] Jiang Z. Y., Chawla A., Bose A., Way M. & Czech M. P. A phosphatidylinositol 3-kinase-independent insulin signaling pathway to N-WASP/Arp2/3/F-actin required for GLUT4 glucose transporter recycling. J. Biol. Chem. 277, 509–515 (2002). 11694514 10.1074/jbc.M108280200

[b45] Bose A. *et al.* Unconventional myosin Myo1c promotes membrane fusion in a regulated exocytic pathway. Mol. Cell. Biol. 24, 5447–5458 (2004). 15169906 10.1128/MCB.24.12.5447-5458.2004 PMC419880

[b46] Chiu T. T., Patel N., Shaw A. E., Bamburg J. R. & Klip A. Arp2/3-and cofilin-coordinated actin dynamics is required for insulin-mediated GLUT4 translocation to the surface of muscle cells. Mol. Biol. Cell 21, 3529–3539 (2010). 20739464 10.1091/mbc.E10-04-0316 PMC2954118

[b47] JeBailey L. *et al.* Skeletal muscle cells and adipocytes differ in their reliance on TC10 and Rac for insulin-induced actin remodeling. Mol. Endocrinol. 18, 359–372 (2004). 14615606 10.1210/me.2003-0294

[b48] Ueda S. *et al.* Crucial role of the small GTPase Rac1 in insulin-stimulated translocation of glucose transporter 4 to the mouse skeletal muscle sarcolemma. FASEB J. 24, 2254–2261 (2010). 20203090 10.1096/fj.09-137380 PMC4183928

[b49] Brozinick J. T., Hawkins E. D., Strawbridge A. B. & Elmendorf J. S. Disruption of cortical actin in skeletal muscle demonstrates an essential role of the cytoskeleton in glucose transporter 4 translocation in insulin-sensitive tissues. J. Biol. Chem. 279, 40699–40706 (2004). 15247264 10.1074/jbc.M402697200 PMC2409066

[b50] JeBailey L. *et al.* Ceramide- and oxidant-induced insulin resistance involve loss of insulin-dependent Rac-activation and actin remodeling in muscle cells. Diabetes 56, 394–403 (2007). 17259384 10.2337/db06-0823

[b51] Marcusohn J., Isakoff S. J., Rose E., Symons M. & Skolnik E. Y. The GTP-binding protein Rac does not couple PI 3-kinase to insulin-stimulated glucose transport in adipocytes. Curr. Biol. 5, 1296–1302 (1995). 8574587 10.1016/s0960-9822(95)00256-9

[b52] Dorrestijn J., Bos J. L., Van Der Zon G. C. M. & Maassen J. A. Changes in the signalling status of the small GTP-binding proteins Rac and Rho do not influence insulin-stimulated hexose transport. Exp. Clin. Endocrinol. Diabetes 105, 254–262 (1997). 9354853 10.1055/s-0029-1211762

[b53] Wang Q. *et al.* Protein kinase B/Akt participates in GLUT4 translocation by insulin in L6 myoblasts. Mol. Cell. Biol. 19, 4008–4018 (1999). 10330141 10.1128/mcb.19.6.4008 PMC104360

[b54] Nozaki S. *et al.* Akt2 regulates Rac1 activity in the insulin-dependent signaling pathway leading to GLUT4 translocation to the plasma membrane in skeletal muscle cells. Cell. Signal. 25, 1361–1371 (2013). 23499910 10.1016/j.cellsig.2013.02.023

[b55] Takenaka N. *et al.* A critical role of the small GTPase Rac1 in Akt2-mediated GLUT4 translocation in mouse skeletal muscle. FEBS J. 281, 1493–1504 (2014). 24438685 10.1111/febs.12719

[b56] Sano H. *et al.* Rab10, a target of the AS160 Rab GAP, is required for insulin-stimulated translocation of GLUT4 to the adipocyte plasma membrane. Cell Metab. 5, 293–303 (2007). 17403373 10.1016/j.cmet.2007.03.001

[b57] Eguez L. *et al.* Full intracellular retention of GLUT4 requires AS160 Rab GTPase activating protein. Cell Metab. 2, 263–272 (2005). 16213228 10.1016/j.cmet.2005.09.005

[b58] Yamashiro S. *et al.* Differential actin-regulatory activities of tropomodulin1 and tropomodulin3 with diverse tropomyosin and actin isoforms. J. Biol. Chem. 289, 11616–11629 (2014). 24644292 10.1074/jbc.M114.555128 PMC4002072

[b59] Meberg P. J., Ono S., Minamide L. S., Takahashi M. & Bamburg J. R. Actin depolymerizing factor and cofilin phosphorylation dynamics: response to signals that regulate neurite extension. Cell Motil. Cytoskeleton 39, 172–190 (1998). 9484959 10.1002/(SICI)1097-0169(1998)39:2<172::AID-CM8>3.0.CO;2-8

[b60] Nishita M. *et al.* Phosphoinositide 3-kinase-mediated activation of cofilin phosphatase Slingshot and its role for insulin-induced membrane protrusion. J. Biol. Chem. 279, 7193–7198 (2004). 14645219 10.1074/jbc.M312591200

[b61] Moyer J. D. *et al.* Tropomodulin 1-null mice have a mild spherocytic elliptocytosis with appearance of tropomodulin 3 in red blood cells and disruption of the membrane skeleton. Blood 116, 2590–2599 (2010). 20585041 10.1182/blood-2010-02-268458 PMC2953891

[b62] Bryce N. S. *et al.* Specification of actin filament function and molecular composition by tropomyosin isoforms. Mol. Biol. Cell 14, 1002–1016 (2003). 12631719 10.1091/mbc.E02-04-0244 PMC151575

[b63] Creed S. J., Desouza M., Bamburg J. R. & Gunning P. Tropomyosin isoform 3 promotes the formation of filopodia by regulating the recruitment of actin-binding proteins to actin filaments. Exp. Cell Res. 317, 249–261 (2011). 21036167 10.1016/j.yexcr.2010.10.019

[b64] Mudry R. E., Perry C. N., Richards M., Fowler V. M. & Gregorio C. C. The interaction of tropomodulin with tropomyosin stabilizes thin filaments in cardiac myocytes. J. Cell Biol. 162, 1057–1068 (2003). 12975349 10.1083/jcb.200305031 PMC2172850

[b65] Gunning P. W., Schevzov G., Kee A. J. & Hardeman E. C. Tropomyosin isoforms: divining rods for actin cytoskeleton function. Trends Cell Biol. 15, 333–341 (2005). 15953552 10.1016/j.tcb.2005.04.007

[b66] Tojkander S. *et al.* A molecular pathway for myosin II recruitment to stress fibers. Curr. Biol. 21, 539–550 (2011). 21458264 10.1016/j.cub.2011.03.007

[b67] Woody S., Stall R., Ramos J. & Patel Y. M. Regulation of myosin light chain kinase during insulin-stimulated glucose uptake in 3T3-L1 adipocytes. PLoS ONE 8, e77248 (2013). 24116218 10.1371/journal.pone.0077248 PMC3792908

[b68] Yang W. *et al.* Regulation of adipogenesis by cytoskeleton remodelling is facilitated by acetyltransferase MEC-17-dependent acetylation of α-tubulin. Biochem. J. 449, 605–612 (2013). 23126280 10.1042/BJ20121121 PMC5573127

[b69] Saito K. *et al.* An enzymatic photometric assay for 2-deoxyglucose uptake in insulin-responsive tissues and 3T3-L1 adipocytes. Anal. Biochem. 412, 9–17 (2011). 21262191 10.1016/j.ab.2011.01.022

[b70] Gonzalez E. & McGraw T. E. Insulin signaling diverges into Akt-dependent and -independent signals to regulate the recruitment/docking and the fusion of GLUT4 vesicles to the plasma membrane. Mol. Biol. Cell 17, 4484–4493 (2006). 16914513 10.1091/mbc.E06-07-0585 PMC1635362

