# Supplementary material for: Tropomodulin3 is a novel Akt2 effector regulating insulin-stimulated GLUT4 exocytosis through cortical actin remodeling
Source: Nat Commun. 2015 Jan 9;6:5951. doi: 10.1038/ncomms6951 (PMC4354152; doi:10.1038/ncomms6951)
Supplement: Supplementary Information — Supplementary Figures 1-7, Supplementary Tables 1-2 and Supplementary References [file ncomms6951-s1.pdf]

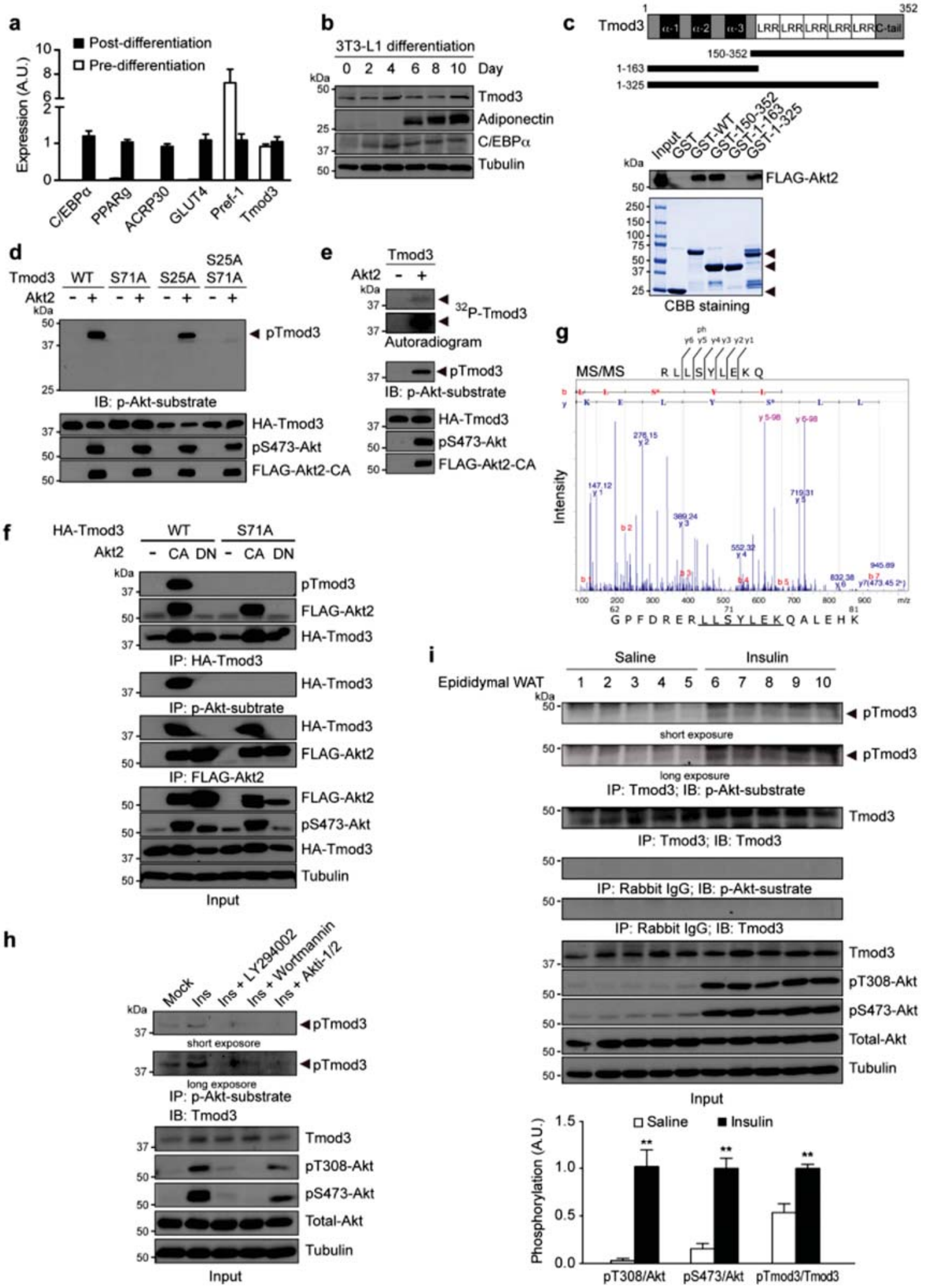
a
Post-differentiation
Pre-differentiation
Expression (A.U.)
C/EBPα
PPARg
ACRP30
GLUT4
Pref-1
Tmod3
b
3T3-L1 differentiation
0 2 4 6 8 10 Day
kDa
Tmod3
Adiponectin
C/EBPα
Tubulin
c
Tmod3
α-1 α-2 α-3 LRR LRR LRR LRR LRR C-tail
1
352
150-352
1-163
1-325
Input GST GST-WT GST-150-352 GST-1-163 GST-1-325
FLAG-Akt2
CBB staining
d
Tmod3 WT S71A S25A S25A S71A
Akt2
pTmod3
IB: p-Akt-substrate
HA-Tmod3
pS473-Akt
FLAG-Akt2-CA
e
Tmod3
Akt2
32P-Tmod3
Autoradiogram
pTmod3
IB: p-Akt-substrate
HA-Tmod3
pS473-Akt
FLAG-Akt2-CA
f
HA-Tmod3 WT S71A
Akt2 - CA DN - CA DN
pTmod3
FLAG-Akt2
HA-Tmod3
IP: HA-Tmod3
HA-Tmod3
IP: p-Akt-subtrate
HA-Tmod3
FLAG-Akt2
IP: FLAG-Akt2
FLAG-Akt2
pS473-Akt
HA-Tmod3
Tubulin
Input
g
MS/MS
R L L S Y L E K Q
Intensity
G P F D R E R L L S Y L E K Q A L E H K
h
Mock
Ins
Ins + LY294002
Ins + Wortmannin
Ins + Akti-1/2
pTmod3
short exposore
long exposore
IP: p-Akt-substrate
IB: Tmod3
Tmod3
pT308-Akt
pS473-Akt
Total-Akt
Tubulin
Input
i
Saline
Insulin
Epididymal WAT 1 2 3 4 5 6 7 8 9 10
pTmod3
short exposure
long exposure
IP: Tmod3; IB: p-Akt-substrate
Tmod3
IP: Tmod3; IB: Tmod3
IP: Rabbit IgG; IB: p-Akt-sustrate
IP: Rabbit IgG; IB: Tmod3
Tmod3
pT308-Akt
pS473-Akt
Total-Akt
Tubulin
Input
Saline
Insulin
Phosphorylation (A.U.)
pT308/Akt
pS473/Akt
pTmod3/Tmod3

Supplementary Figure 1 | Tmod3 expression and phosphorylation. (a) Expression of Tmod3 in 3T3-L1 preadipocytes and differentiated adipocytes. Expression of various genes in both 3T3-L1 preadipocytes and differentiated adipocytes was analyzed by real-time quantitative PCR (qPCR). n = 7 per group. **(b)** Protein expression at different stages of 3T3-L1 differentiation was examined from day 0 to day 10. Tubulin was used as a loading control. **(c)** Binding of Tmod3 to Akt2 requires LRR domains. GST-fused full length or truncation mutants of Tmod3 were incubated with lysates of HEK293T cells expressing FLAG-Akt2-WT. **(d)** Ser71 is the phosphorylation target of Akt2 as demonstrated by *in vitro* Akt2 kinase assay. **(e)** *In vitro* kinase assay showing Tmod3 phosphorylation by Akt2. Purified full-length mouse Tmod3 was incubated with constitutively active Akt2 and [^{32}P]γ-ATP. The samples were analyzed by SDS-PAGE and autoradiography. **(f)** Tmod3 interacts with constitutively active Akt2, but not kinase-dead Akt2, and becomes phosphorylated. Akt2-mediated phosphorylation of Tmod3 in HEK293T cells following co-expression of Tmod3 (WT or S71A) with either constitutively active (CA) or kinase-dead mutant (DN) of Akt2. **(g)** Identification of Ser71 at α-helix2 as target phosphorylation site by mass spectrometry. **(h)** Insulin induces phosphorylation of Tmod3 in a PI3K/Akt signaling dependent manner. Anti-PAS antibody was used to pull down phosphorylated Akt substrates from cell lysates followed by immunoblotting with Tmod3 Antibody. **(i)** Insulin induces phosphorylation of Tmod3 *in vivo*. After 4-hour fasting, 12-weeks old C57BL/6 male mice were injected i.p. with saline or insulin (1 U/kg body weight). Fifteen minutes after injection, mice were sacrificed and epididymal white adipose tissues (WAT) were homogenized. Tmod3 antibody pre-conjugated to protein A/G Sepharose beads was used to immune-precipitate Tmod3 from tissue lysates. Data are expressed as mean ± SD (n = 5 per group; unpaired Student's *t*-test). **$p < 0.01$ versus insulin treatment.

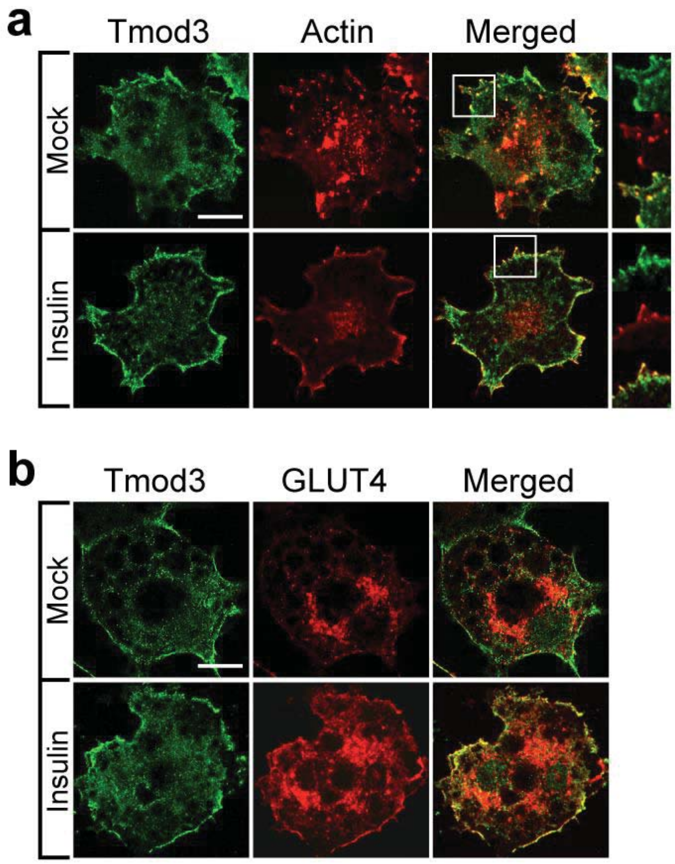

Supplementary Figure 2 | Co-localization of Tmod3 with F-actin and GLUT4. (a) Cells were immunolabeled for FLAG-Tmod3 (green) and actin filaments were stained with Alexa Fluor-568-Phalloidin (red). The side panels show enlarged views of the boxed regions. Scale bar: 20 μm. **(b)** Co-localization of FLAG-Tmod3 and GLUT4-mCherry at the periphery in adipocytes in response to insulin stimulation. Scale bar: 20 μm. The images shown were taken at the ventral section of adipocytes by confocal microscopy.

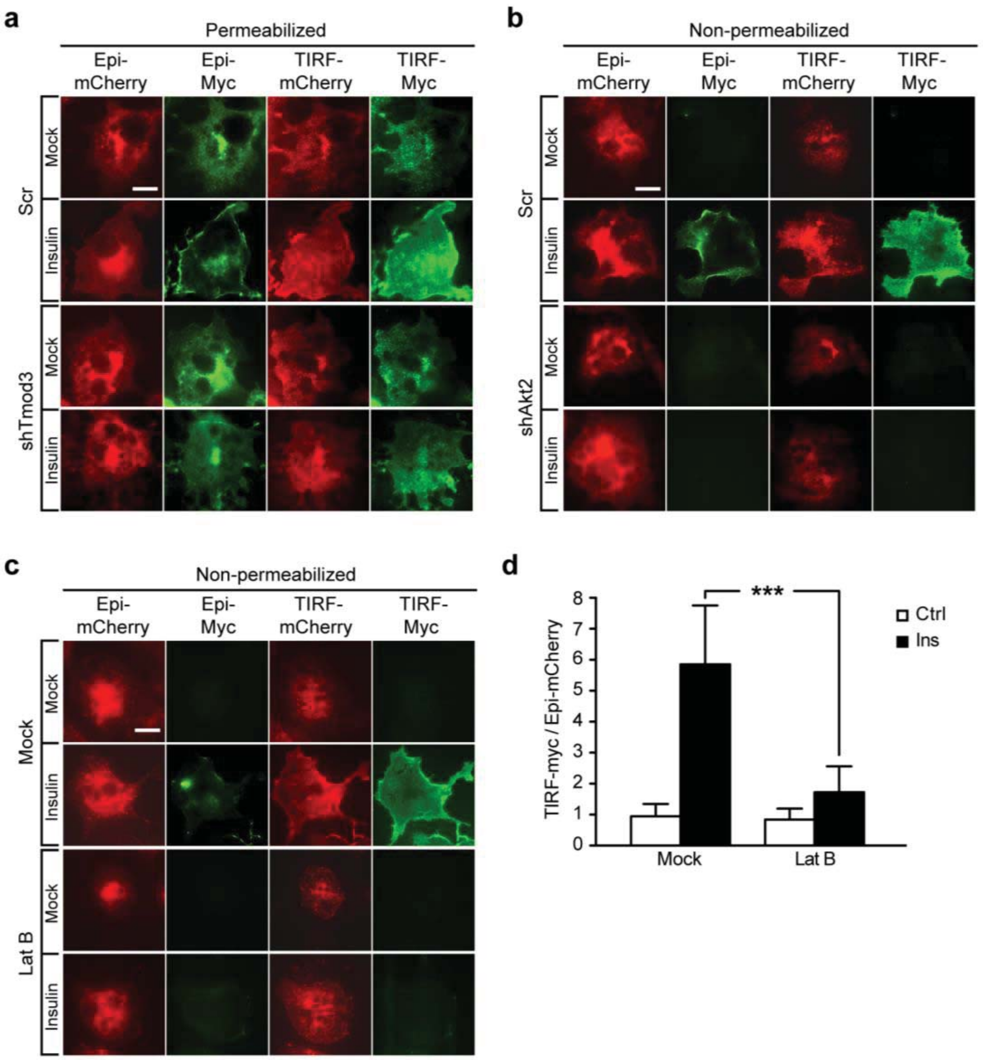

Supplementary Figure 3 | Impaired GLUT4 exocytosis in Akt2-KD adipocytes. (a) Fixed cells were permeabilized with saponin followed by immunolabeling with anti-Myc antibody and AlexaFluor-488 dye. Permeabilized samples were imaged by TIRFM. Scale bar: 20 μm. **(b)** Both TIRF-mCherry and TIRF-Myc signals were detected in insulin-stimulated scrambled control cells in TIRF zone, indicating proper GLUT4 translocation and fusion with the PM. However, TIRF signals were significantly decreased in Akt2-deficient adipocytes. Scale bar: 20 μm. **(c,d)** Latrunculin B inhibits vesicle fusion but not trafficking of GLUT4 to the cell periphery. 3T3-L1 adipocytes expressing myc-GLUT4-mCherry were serum-starved for 2 hours and pre-treated with 10 μM Latrunculin B for 30 min followed by 10 nM insulin treatment for 20 min at 37°C. Cells were fixed and labeled by anti-Myc antibody followed by Alexa Fluor 488-conjugated goat anti-mouse secondary antibody (green) under non-permeabilized condition and then imaged by TIRFM. Data are shown as the ratio of cell surface TIRF-Myc signal to total Epi-mCherry and presented as mean ± SD (n = 30 cells per group; ANOVA with Dunnett's multiple comparison test). ***p < 0.0001 versus Mock Insulin groups. Representative microscopic images are shown in **(c)**. Scale bar: 20 μm.

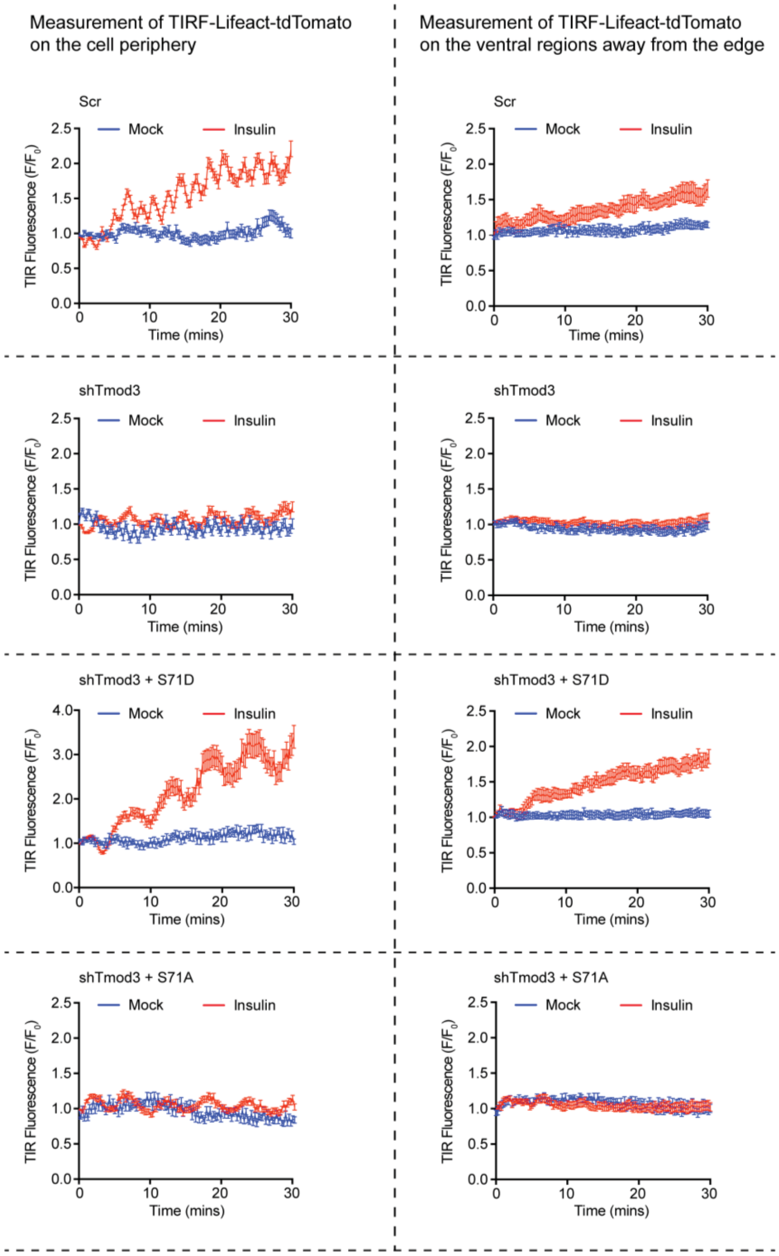

Supplementary Figure 4 | Representative examples of TIRFM-Lifeact-tdTomato actin remodeling analysis. Cells were serum-starved, treated with or without insulin and imaged for 30 mins at an interval of 15 seconds. Measurement on the cell periphery: after removal of background fluorescence, TIRF intensities of all ROIs measured over time are normalized to the intensity measured at zero time point, averaged, and plotted against the time to indicate the time course of actin remodeling. n = 6-8 cells per condition were analyzed. Analyses of representative cells are shown in **(a)** Scr, **(b)** shTmod3, **(c)** shTmod3 + S71D, and **(d)** shTmod3 + S71A. Original movies for these images are in Supplementary Movies 1a-b, 2a-f. Measurement on the ventral regions away from the edge: n = 8-17 cells per condition for each group were included for analysis. For details of the analysis, please refer to the Methods section.

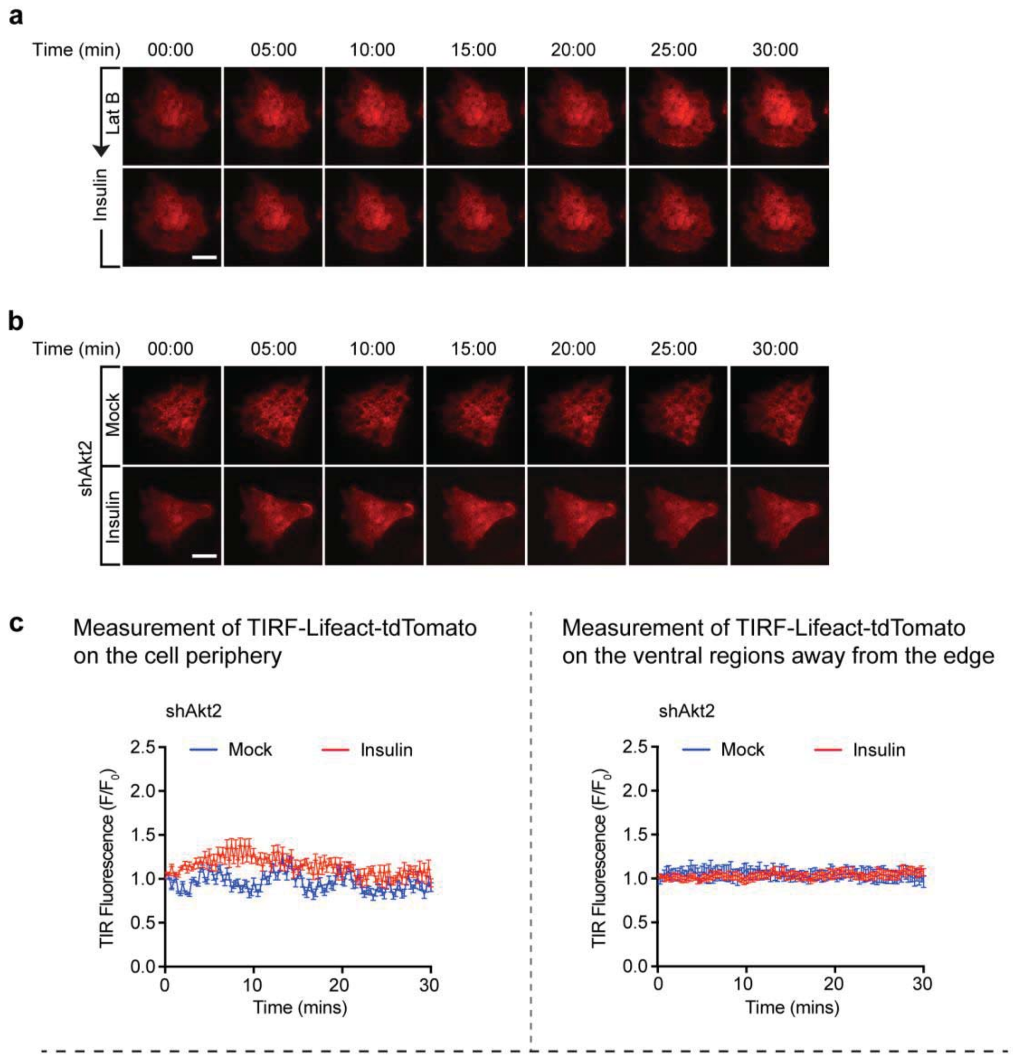

Supplementary Figure 5 | Actin remodeling in adipocytes under Latrunculin B treatment and in Akt2-KD cells. (a) Selected time-lapse images of actin remodeling in a representative cell expressing Lifeact-tdTomato under Latrunculin B treatment and followed by insulin stimulation for another 30 min. Original movies for these images are in Supplementary Movies 1c-d. **(b)** Selected time-lapse images of actin remodeling in Akt2-KD adipocytes expressing Lifeact-tdTomato under insulin stimulation. Original movies for these images are in Supplementary Movies 2g-h. **(c)** Representative analysis of TIRFM-based actin remodeling in Akt2 KD adipocytes. Measurement on the cell periphery: after removal of background fluorescence, TIRF intensities of all ROIs measured over time were normalized to the intensity measured at zero time point, averaged, and plotted against the time to indicate the time course of actin remodeling. n = 6-8 cells per condition were analyzed. Measurement on the ventral regions away from the edge: n = 8-17 cells per condition for each group were included for analysis.

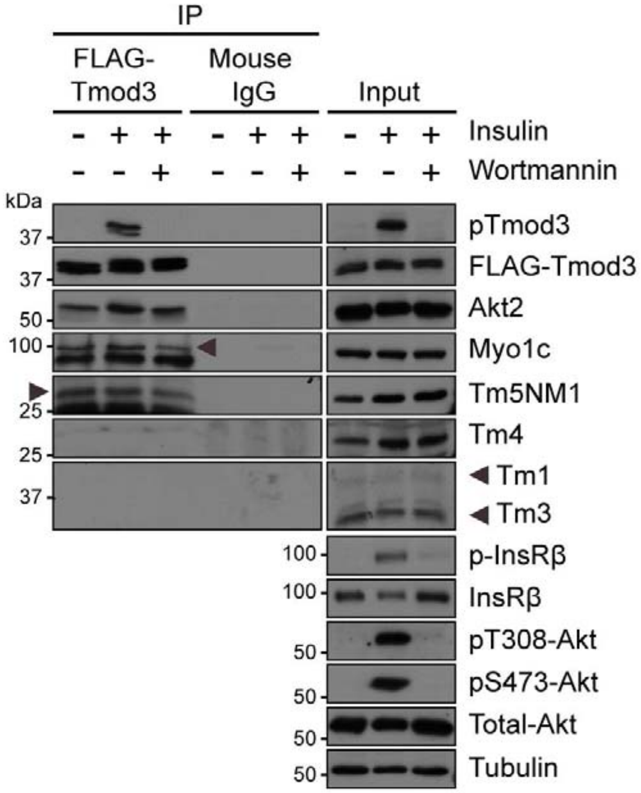

Supplementary Figure 6 | Tm5NM1 is the specific Tmod3-binding tropomyosin isoform in adipocytes. After 2-hr serum starvation, 3T3-L1 adipocytes expressing FLAG-Tmod3 received mock, insulin, or insulin plus wortmannin treatments. Tmod3 immunoprecipitates were analyzed by immunoblotting to identify specific Tmod3-interacting tropomyosin isoform.

Supplementary Figure 7 | Uncropped scans of important blots and gels used in figures with highlighted boxes.

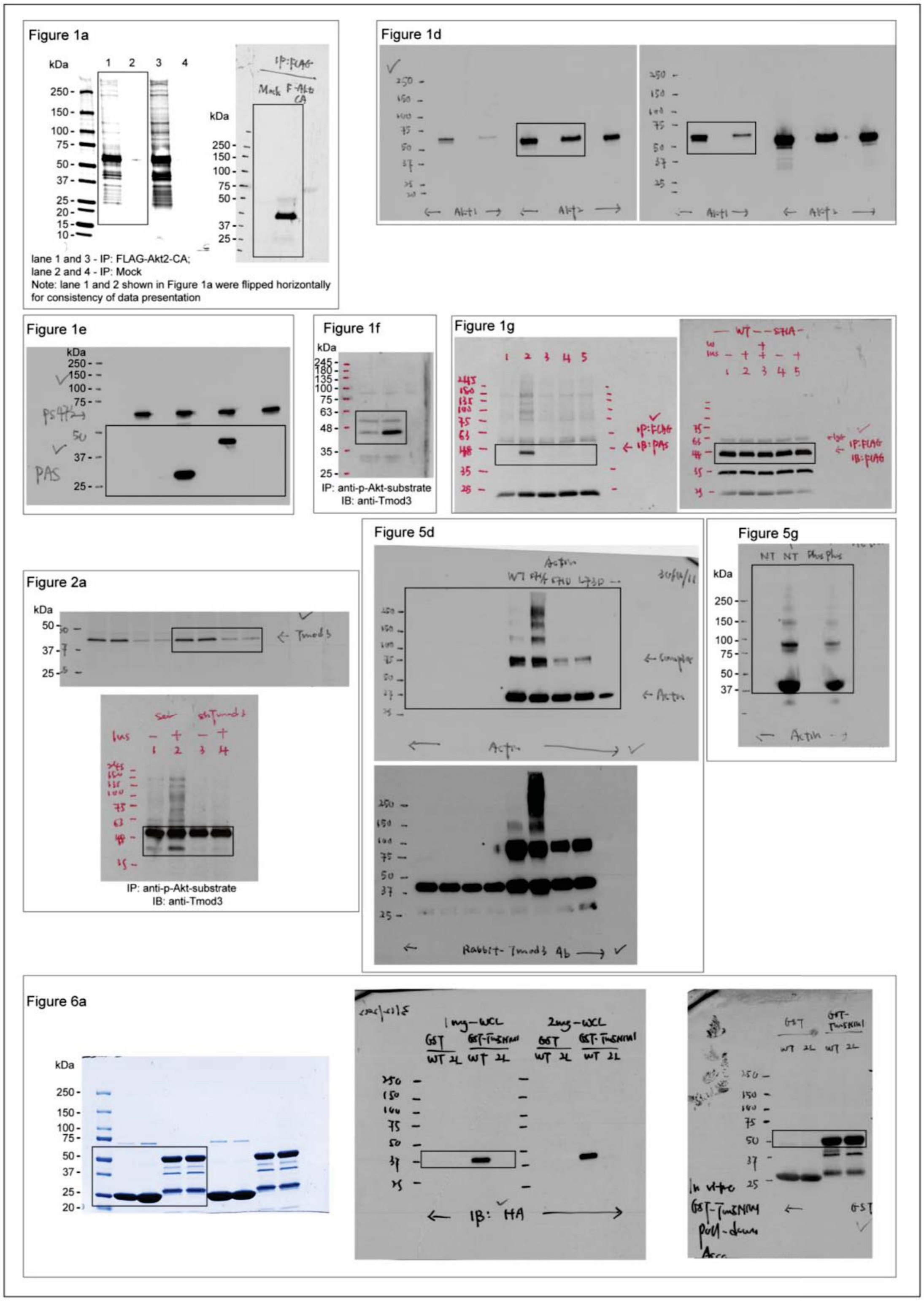

Supplementary Table 1 | A list of antibodies used in the study

**Antibodies**	**Isotype**	**Use**	**Product No.**	**Source**
Anti-FLAG M2 Affinity Gel	Mouse	IP (10 µl/mg)	A2220	Sigma
Anti-FLAG Ab	Mouse	WB (1:3000)<br>IF (1:500)	F1804	Sigma
EZview Red Anti-HA Affinity Gel	Mouse	IP (10 µl/mg)	E6779	Sigma
Anti-Tubulin	Mouse	WB (1:10000)	T5168	Sigma
Anti-HA	Mouse	WB (1:3000)	3724	Cell Signaling
Anti-Akt	Rabbit	WB (1:1000)	9272	Cell Signaling
Anti-pS473-Akt	Rabbit	WB (1:1000)	9271	Cell Signaling
Anti-pT308-Akt	Rabbit	WB (1:1000)	9275	Cell Signaling
Anti-Akt1	Rabbit	WB (1:1000)	2967	Cell Signaling
Anti-Akt2	Rabbit	WB (1:1000)	3063	Cell Signaling
Anti-phospho-Akt-substrate (PAS)	Rabbit	WB (1:1000)	9614	Cell Signaling
Anti-Tropomyosin1/3	Rabbit	WB (1:1000)	3913	Cell Signaling
Anti-IRAP	Rabbit	WB (1:1000)	6918	Cell Signaling
Anti-C/EBPα	Rabbit	WB (1:1000)	9314	Santa Cruz
Anti-adiponectin	Rabbit	WB (1:1000)	sc-17044	Santa Cruz
Anti-GST	Mouse	WB (1:3000)	sc-138	Santa Cruz
Anti-Myc, clone 9E10	Mouse	WB (1:3000)<br>IF (1:500)	sc-40	Santa Cruz
Anti-Tmod3	Rabbit	WB (1:1000)<br>IP (2 µg/mg)	ARP55078	AVIVA
Anti-γTm9d for detection of Tm5NM1	Mouse	WB (1:1000)		P.W. Gunning[1]
Anti-δTm9d for detection of Tm4	Rabbit	WB (1:1000)		
Anti-CG1 for detection of Tm1	Mouse	WB (1:1000)		
Anti-αTm9d for detection of Tm1,2,3,5a,5b &6	Mouse	WB (1:1000)		

Supplementary Table 2 | A list of plasmids used in the study

Plasmids	Sense/Antisense primers	Home-made unless otherwise stated
pCMV5-HA/FLAG-mTmod3-WT	5'-GGA ATT CAG ATG GCA CTG CCG TTC CGG AAG-3'<br>5'CGG GAT CCC GTT ACT GGT GGT CTC CTT CAA TTC G-3'	5'-EcoRI; 3'-BamHI<br>Mouse 3T3-L1 cDNA library
pCMV5-HA/FLAG-mTmod3-S71A	5'-GAG AAC GGC TCC TTG CTT ACC TAG AGA AG-3'<br>5'-CTT CTC TAG GTA AGC AAG GAG CCG TTC TC-3'	Site-directed mutagenesis. phospho-defective mutant
pCMV5-HA/FLAG-mTmod3-S71D	5'-GAG AAC GGC TCC TTG ACT ACC TAG AGA AG-3'<br>5'-CTT CTC TAG GTA GTC AAG GAG CCG TTC TC-3'	Site-directed mutagenesis. phospho-mimetic mutant
pCMV5-HA/FLAG-mTmod3-L73D	5'-GAA CGG CTC CTT TCT TAC GAT GAG AAG CAA GCA TTG GAG-3'<br>5'-CTC CAA TGC TTG CTT CTC ATC GTA AGA AAG GAG CCG TTC-3'	Site-directed mutagenesis. loss of G-actin binding
pCMV5-HA/FLAG-mTmod3-S25A	5'-CTT GGC AAG CTG GCC GAA TCA GAG C-3'<br>5'-GCT CTG ATT CGG CCA GCT TGC CAA G-3'	Site-directed mutagenesis.
pCMV5-HA/FLAG-mTmod3-L29G	5'-CTG TCC GAA TCA GAG GGG AAA CAG CTG GAG AC-3'<br>5'-GTC TCC AGC TGT TTC CCC TCT GAT TCG GAC AG-3'	Site-directed mutagenesis. defective in Tm-binding
pCMV5-HA/FLAG-mTmod3-L134D	5'-CAG AGC TGT GCG ACG ATG CAG CTA TTC TTG G-3'<br>5'-CCA AGA ATA GCT GCA TCG TCG CAC AGC TCT G-3'	Site-directed mutagenesis. defective in Tm-binding
pGEX-KG-mTmod3-series	5'-GGA ATT CAG ATG GCA CTG CCG TTC CGG AAG-3'<br>5'-CGG TTA ACG TTA CTG GTG GTC TCC TTC AAT TCG-3'	PCR→EcoRI + HpaI cut, ligate into pGEX-KG-EcoRI + (XhoI)
pGEX-KG-mTmod3-1-163	5'-GGA ATT CAG ATG GCA CTG CCG TTC CGG AAG-3'<br>5'-CGG TTA ACG CTA ACG CTC TTG GTT GAC ACC G-3'	PCR→EcoRI + HpaI cut, ligate into pGEX-KG-EcoRI + (XhoI)
pGEX-KG-mTmod3-150-352	5'-CGG AGG AAT TCA GTG TGA TGT GCT GGG AAG-3'<br>5'-CGG TTA ACG TTA CTG GTG GTC TCC TTC AAT TCG-3'	PCR→EcoRI + HpaI cut, ligate into pGEX-KG-EcoRI + (XhoI)
pGEX-KG-mTmod3-1-325	5'-GGA ATT CAG ATG GCA CTG CCG TTC CGG AAG-3'<br>5'-CGG TTA ACG CTA CTG CTG CGT GAA CTG ATA TC-3'	PCR→EcoRI + HpaI cut, ligate into pGEX-KG-EcoRI + (XhoI)

pCMV5-FLAG-mTmod3-RR	5'-GGG AAA ACG ATG CTC ATC TTG TTG AAG-3'<br>5'-CTT CAA CAA GAT GAG CAT CGT TTT CCC-3'	Site-directed mutagenesis. Resistance to pLKO.1-shTmod3
pLenti-FLAG-mTmod3-series	5'-CGC GCT AGC GCC ATG GAC TAC AAG GAC GAT-3'<br>5'-CGG TTA ACG TTA CTG GTG GTC TCC TTC AAT TCG-3'	PCR→NheI + HpaI cut, ligate into pLenti-hiko-NheI + HpaI
pLKO.1-shTmod3	5'-CCG GGC CCG TCT TGT TGA AGT TAA TCT CGA GAT TAA CTT CAA CAA GAC GGG CTT TTT G-3'<br>5'-AAT TCA AAA AGC CCG TCT TGT TGA AGT TAA TCT CGA GAT TAA CTT CAA CAA GAC GGG C-3'	shRNA against mouse Tmod3
pLKO.1-scrambled	5'-CCG GAC AAC AGC CAC AAC GTC TAT ACT CGA GTA TAG ACG TTG TGG CTG TTG TTT TTT G-3'<br>5'-AAT TCA AAA AAC AAC AGC CAC AAC GTC TAT ACT CGA GTA TAG ACG TTG TGG CTG TTG T-3'	shRNA against GFP
pcDNA3-FLAG-HA-Akt1		Human Akt1<br>Addgene plasmid 9021[2]
pcDNA3-myr-HA-Akt2		Human Akt2<br>Addgene plasmid 9016
pCMV5-FLAG-Akt2-WT		pcDNA3-myr-HA-Akt2-(BamHI) + XbaI cut, ligate into pCMV5-FLAG-(EcoRI) +XbaI
pCMV5-FLAG-Akt2-DN R274H	5'-GAC GTG GTA TAC CAC GAC ATC AAG CTG G-3'<br>5'-CCA GCT TGA TGT CGT GGT ATA CCA CGT C-3'	Site-directed mutagenesis. kinase inactive mutant[1,3]
pLenti-FLAG-Akt2-series	5'-CGC GCT AGC GCC ATG GAC TAC AAG GAC GAT-3'<br>5'-GCG TTA ACG AAT TCT CAC TCG CGG ATG CTG GC-3'	PCR→NheI + HpaI cut, ligate into pLenti-hiko-NheI + HpaI
pLenti-FLAG-Akt2-CA (myr-FLAG-Akt2)	5'-AAT TGG CCA TGG GTT CCT CCA AAT CCA AGC CCA AGG-3'<br>5'-CTA GCC TTG GGC TTG GAT TTG GAG GAA CCC ATG GCC-3'	Annealing oligos carrying myr sequence-5'-EcoRI/3'-NheI into pLenti-FLAG-Akt2
pMyc-GLUT4-mCherry	5'-CGC GAA GCC GAA GAA CAG AAA CTG ATC TCT GAA GAA GAC CTG CTG AAG-3'<br>5'-CGC GCT TCA GCA GGT CTT CTT CAG AGA TCA GTT TCT GTT CTT CGG CTT-3'	Annealing oligos carrying Myc sequence; MluI site insertion into sequence encoding 1st exofacial loop of pGLUT4-mCherry

pLenti-Myc-GLUT4-mCherry		pMyc-GLUT4-mCherry-NheI + HpaI cut, ligate into pLenti-hiko-NheI + HpaI
pLifeact-Tdtomato	5’-AAT TCA TGG GCG TGG CCG ACC TGA TCA AGA AGT TCG AGA GCA TCA GCA AGG AGG AG-3’<br>5’-CCG GCT CCT CCT TGC TGA TGC TCT CGA ACT TCT TGA TCA GGT CGG CCA CGC CCA TG-3’	Annealing oligos carrying Lifeact sequence – EcoRI + AgeI insertion into pTdtomato-N1[1,2,4]
pLenti-Lifeact-Tdtomato		pLifeact-Tdtomato-N1-NheI + HpaI cut, ligate into pLenti-hiko vector-NheI + HpaI

Supplementary References

1. Schevzov, G., Whittaker, S. P., Fath, T., Lin, J. J. & Gunning, P. W. Tropomyosin isoforms and reagents. *Bioarchitecture* **1,** 135–164 (2011).
2. Hsieh, A. C. *et al.* A library of siRNA duplexes targeting the phosphoinositide 3-kinase pathway: determinants of gene silencing for use in cell-based screens. *Nucleic Acids Res* **32,** 893–901 (2004).
3. George, S. *et al.* A family with severe insulin resistance and diabetes due to a mutation in AKT2. *Science* **304,** 1325–1328 (2004).
4. Riedl, J. *et al.* Lifeact: a versatile marker to visualize F-actin. *Nat Methods* **5,** 605–607 (2008).
